# Effects of Exercise in the Treatment of Overweight and Obese Children and Adolescents: A Systematic Review of Meta-Analyses

**DOI:** 10.1155/2013/783103

**Published:** 2013-12-24

**Authors:** George A. Kelley, Kristi S. Kelley

**Affiliations:** ^1^Meta-Analytic Research Group, Department of Biostatistics, School of Public Health, Robert C. Byrd Health Sciences Center, West Virginia University, Morgantown, WV 26506-9190, USA; ^2^Department of Biostatistics, School of Public Health, Robert C. Byrd Health Sciences Center, West Virginia University, P.O. Box 9190, Morgantown, WV 26506-9190, USA

## Abstract

*Purpose*. Conduct a systematic review of previous meta-analyses addressing the effects of exercise in the treatment of overweight and obese children and adolescents. *Methods*. Previous meta-analyses of randomized controlled exercise trials that assessed adiposity in overweight and obese children and adolescents were included by searching nine electronic databases and cross-referencing from retrieved studies. Methodological quality was assessed using the Assessment of Multiple Systematic Reviews (AMSTAR) Instrument. The alpha level for statistical significance was set at *P* ≤ 0.05. *Results*. Of the 308 studies reviewed, two aggregate data meta-analyses representing 14 and 17 studies and 481 and 701 boys and girls met all eligibility criteria. Methodological quality was 64% and 73%. For both studies, statistically significant reductions in percent body fat were observed (*P* = 0.006 and *P* < 0.00001). The number-needed-to treat (NNT) was 4 and 3 with an estimated 24.5 and 31.5 million overweight and obese children in the world potentially benefitting, 2.8 and 3.6 million in the US. No other measures of adiposity (BMI-related measures, body weight, and central obesity) were statistically significant. *Conclusions*. Exercise is efficacious for reducing percent body fat in overweight and obese children and adolescents. Insufficient evidence exists to suggest that exercise reduces other measures of adiposity.

## 1. Introduction

Worldwide, the prevalence of overweight and obesity has reached epidemic proportions and includes not only adults [1] but also children and adolescents [2, 3]. For example, it has been reported that the worldwide prevalence of overweight and obesity includes approximately 110 million children [2] while in the United States (US), an estimated 12.5 million children and adolescents are either overweight or obese [4]. This is problematic because overweight and obese youth have been shown to be at an increased risk of becoming overweight and obese adults [5], and thus placing them at an increased risk for premature all-cause mortality [6]. Based on 2005 data, overweight and obesity as well as physical inactivity in adults were reported to be the third leading causes of preventable death in the US (about 1 in 10 deaths each) behind cigarette smoking and high blood pressure [6]. The issue of obesity has become so problematic that it has recently been recognized as a disease by the American Medical Association [7].

Exercise, a nonpharmacologic intervention that is available to the vast majority of the general public, may play a pivotal role in the treatment of overweight and obese children and adolescents. Systematic reviews with meta-analysis, a quantitative approach for combining the results of different studies on the same topic [8], are considered by many to be the most important type of evidence for determining the efficacy and effectiveness of various treatments on selected outcomes [9, 10]. Unfortunately, with the proliferation of systematic reviews on the same topic, it becomes difficult to make informed decisions regarding the effects of various interventions on selected outcomes. For example, a recent systematic review identified 22 previous meta-analyses examining the effects of exercise on blood pressure [11]. Given the proliferation of reviews, a need now exists to systematically review these previous reviews in order to provide decision-makers with the information they need to make evidence-based decisions regarding the efficacy and effectiveness of various interventions on selected outcomes as well as provide direction for future research [12]. Given the former, the purpose of the current study was to conduct a systematic review of previous meta-analyses addressing the effects of exercise (aerobic, strength training, or both) in the treatment of overweight and obese children and adolescents.

## 2. Methods

### 2.1. Study Eligibility

The *a priori *inclusion criteria for this study were as follows: (1) previous systematic reviews with meta-analysis of randomized controlled trials or data reported separately for randomized controlled trials, (2) children and adolescents 5 to 18 years of age, (3) aerobic exercise and/or progressive resistance training intervention(s) lasting for an average of at least 4 weeks, (4) published and unpublished (dissertations and master's theses) studies in any language from 1990 forward, and (5) exercise minus control group difference in one or more of the following variables that were primary outcomes in the original meta-analysis: body weight, body mass index, body mass index percentile, body mass index *z*-score, percent body fat, fat mass, and fat-free mass. *Post hoc, *percentage overweight, adjusted for height as well as waist-to-hip ratio were also included as outcomes. Meta-analyses were limited to randomized controlled trials because they are the only way to control for unknown confounders as well as the fact that nonrandomized controlled trials tend to overestimate the effects of treatment in healthcare interventions [13, 14]. Potentially eligible meta-analyses were also limited to those that included studies in which exercise was an intervention, defined here as “planned, structured, and repetitive and purposive in the sense that the improvement or maintenance of one or more components of physical fitness is the objective” [15]. While somewhat arbitrary, 4 weeks was chosen as the minimum length of exercise since one should expect some type of change in overweight/obese outcomes during this period of time [16]. Based on a PubMed search, 1990 was chosen as the starting point for searching because it was the first year in which a potentially eligible study was identified for review [17]. Any studies that did not meet all of the above criteria were excluded from our review. Ineligible studies were broadly categorized as excluded based on one or more of the following reasons: (1) inappropriate population (adults, animals, etc.), (2) inappropriate intervention (nutrition, pharmacologic, etc.), (3) inappropriate comparison (exercise versus diet), (4) inappropriate outcome (blood pressure, lipids, etc.), and (5) inappropriate study type (meta-analysis that included nonrandomized controlled trials, systematic review without meta-analysis, etc.).

### 2.2. Data Sources

Using the graphical-user interfaces for each database, the following electronic sources were searched: (1) PubMed, (2) Sport Discus, (3) Web of Science, (4) Scopus, (5) ProQuest, (6) Cochrane Database of Systematic Reviews (CDSR), (7) Physiotherapy Evidence Database (PEDRO), (8) Database of Abstract of Reviews of Effects (DARE), and (9) Health Evidence Canada (HEC). All searches were conducted during the month of April, 2013 with the last searches conducted on April 20. Scopus was included in our database searches because it has been reported to provide coverage of Embase [18]. With the exception of PubMed, which was searched from its inception in order to identify a starting year for searching, all other databases were searched from 1990 forward. A list of all search strategies for each database is shown in Supplementary File 1 (Supplementary Material available online at http://dx.doi.org/10.1155/2013/783103). In addition to electronic database searches, cross-referencing for potentially eligible meta-analyses from retrieved reviews was also conducted. All studies were stored in Reference Manager, version 12.0 [19].

### 2.3. Study Selection

All studies were selected by both authors, independent from each other. They then met and reviewed their selections for agreement. Any disagreements were resolved by consensus.

### 2.4. Data Abstraction

Prior to data abstraction, coding sheets were developed in Microsoft Excel 2010 [20]. The coding sheets could hold up to 253 items from each included meta-analysis. Both authors coded all studies independent of each other. Upon completion of coding, all coding sheets were merged into one common codebook and reviewed by both authors for correctness. Disagreements were resolved by consensus. Using Cohen's kappa statistic (**κ**) [21], the overall agreement rate prior to correcting discrepancies was 0.68.

A copy of the final codebook is available upon request from the corresponding author.

### 2.5. Methodological Quality

Methodological quality for each included meta-analysis was assessed using the Assessment of Multiple Systematic Reviews (AMSTAR) Instrument [22–25]. AMSTAR was chosen over other instruments [26, 27] because of its reported interrater reliability (*κ* = 0.70), construct validity (intraclass correlation coefficient = 0.84) and feasibility (average of 15 minutes per study to complete) [24]. The 11-item questionnaire is designed to elicit responses of “Yes”, “No”, “Can't Answer”, or “Not Applicable”. The response “Can't Answer” is chosen when an item is relevant but not described. The response “Not Applicable” is chosen when an item is not relevant (meta-analysis of data not possible, etc.) [22–25]. For consistency when summing responses, the following question was modified from “Was the status of publication (i.e. grey literature) used as an inclusion criterion?” to “Was the status of publication (i.e. grey literature) as an inclusion criterion avoided?” In addition, we considered the question regarding conflict of interest as adequately met if the authors of the systematic review provided a statement on conflict of interest versus the reporting of conflict of interest by both the authors of the systematic review and the original studies included in the meta-analysis. Both authors, independent from each other, assessed methodological quality. They then met and reviewed every item for correctness. Disagreements were resolved by consensus. Using Cohen's kappa statistic (**κ**) [21], the overall agreement rate prior to correcting discrepancies was 0.82.

### 2.6. Data Synthesis

*A priori*, the overall results from each meta-analysis were extracted [8], with a focus on random effects models since they incorporate between-study heterogeneity into the model and should almost always be the model of choice regardless of whether or not significant heterogeneity exists [28, 29]. Overall point estimates and 95% confidence intervals (CIs) along with the *Q* statistic, a measure of heterogeneity, were extracted for each outcome. An alpha value ≤0.10 was considered to represent statistically significant heterogeneity [30]. However, because of issues surrounding the power of the *Q* statistic, the *I* ^2^ statistic was also reported if it was provided in the meta-analysis. If it was not provided, it was calculated if sufficient data existed to do so [30]. The *I* ^2^ statistic = 100% ∗ (*Q* − df)/*Q*, where *Q* is Cochran's heterogeneity statistic [31] and df, the degrees of freedom [30]. Negative values of *I* ^2^ are set to zero (0) so that *I* ^2^ falls between 0% and 100% [30]. A value of 0% indicates no observed heterogeneity while larger values indicate increasing heterogeneity [30]. While somewhat arbitrary, values of 25%, 50%, and 75% were considered to represent low, moderate, and high amounts of heterogeneity [30].

Since it was assumed that none of the eligible meta-analyses would include 95% prediction intervals (PIs), these were calculated if the overall findings were statistically significant and the results from each study included in each meta-analysis were provided [32–34]. Prediction intervals are used to estimate the treatment effect in a new trial [32–34] and are calculated as follows:

(1) $$\begin{array}{c} \text{mean}\pm t_{\text{df}}*\sqrt{\left({\text{se}}^{2}+\;\tau^{2}\right)}, \end{array}$$

where *t* is the centile point (95%) of the *t* distribution with *k* − 2 degrees of freedom, se^2^ is the squared standard error, and *τ* ^2^ is the between-study variance [34, 35]. All PIs were calculated using the user-written metan command [35] in version 11.0 of Stata [36].

In order to enhance application, the number-needed-to treat (NNT) was calculated for any overall findings that were reported as statistically significant [37]. In addition, the NNT was used to provide gross estimates of the number of obese children and adolescents in the US who could benefit from exercise, based on 12.5 million obese children and adolescents [4] as well as the number of overweight and obese children worldwide who could benefit from exercise, based on 110 million overweight or obese children [2, 38]. It was assumed that none of the children and adolescents included in the original estimates were exercising regularly.

Because neither of the included meta-analyses assessed publication bias or conducted influence analysis with each outcome deleted from the model once, a *post-hoc* decision was made to test for both if sufficient data were provided. Publication bias was assessed using the regression-intercept approach of Egger et al. [39]. Both publication bias and influence analysis were conducted using Comprehensive Meta-Analysis (version 2.2) [40].

For those outcomes that were reported using the standardized mean difference (SMD), values of 0.2, 0.5, and 0.80, were considered to represent small, medium, and large effects [41]. With the exception of heterogeneity, tests with two-tailed alpha levels ≤0.05 were considered to be statistically significant. Two-tailed alpha levels >0.05 but ≤0.10 were considered as a trend towards statistical significance. Precision of estimates was considered robust if 95% confidence intervals for continuous outcomes did not cross zero (0). Dispersion statistics were reported as either standard deviations (SD) or standard errors (SE). With the exception of fat-free mass, negative values for all other outcomes were indicative of improvement.

## 3. Results

### 3.1. Characteristics of Included Meta-Analyses

**Figure 1:**
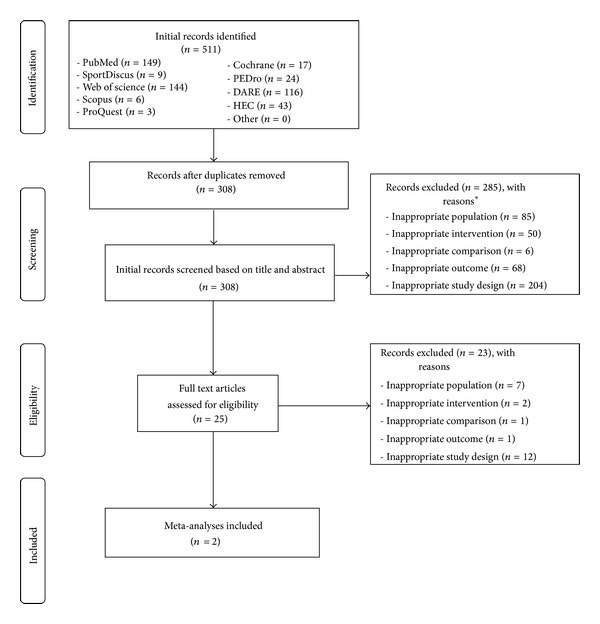
Flow diagram for selection of articles. *Number of reasons exceeds number of records because some records are excluded for more than one reason.

Of the 511 citations initially identified, 308 (60.3%) remained after removing duplicates. Of the 308 articles that were screened, the full text from 25 articles (8.1%) was retrieved and assessed for potential eligibility. Upon completion of the review, two aggregate data meta-analyses met the criteria for inclusion [42, 43]. The major reasons for exclusion of the other studies were an inappropriate study design (49.8%) followed by an inappropriate population (21.2%), outcome (15.9%), intervention (12.0%), and comparison (1.6%). A flow diagram that depicts the search process can be found in Figure 1 while a list of excluded studies, including the reasons for exclusion, is shown in Supplementary File 2. For the two included meta-analyses [42, 43], one focused specifically on exercise [42] while the other focused on nonsurgical interventions, including exercise [43]. Both meta-analyses included overweight and obese children and adolescents according to the criteria described by each of the original studies they included [42, 43]. A general description of the characteristics of each meta-analysis is provided in Table 1.

### 3.2. Methodological Quality

The study by Atlantis et al. [42] satisfied 7 of the 11 AMSTAR criteria (64%) while the study by McGovern et al. [43] satisfied 8 of the 11 criteria (73%). Both meta-analyses were judged as (1) not avoiding the status of publication as an inclusion criterion, (2) not providing a list of excluded studies, and (3) not assessing for potential publication bias [42, 43]. In addition, the meta-analysis by Atlantis et al. [42] was judged as not providing a conflict of interest statement. AMSTAR results for each question from each meta-analysis are shown in Supplementary File 3.

### 3.3. Data Synthesis

#### 3.3.1. Overall Results

A description of the overall findings from each meta-analysis is shown in Table 2. A statistically significant reduction in percent body fat along with nonoverlapping 95% CIs was observed for both the Atlantis et al. [42] (*P* = 0.006) and McGovern et al. [43] (*P* < 0.00001) meta-analyses. Heterogeneity was moderate and statistically significant in the Atlantis et al. [42] but not the McGovern et al. [43] meta-analysis while nonoverlapping 95% PIs were observed for the McGovern et al. [43] but not Atlantis et al. [42] meta-analysis. An examination for publication bias indicated no statistically significant publication bias for either the Atlantis et al. [42] (*b*_0_, −0.32, 95% CI, −4.0 to 3.4, *P* = 0.84) or McGovern et al. [43] (*b*_0_, −1.09, 95% CI, −3.4 to 1.2, *P* = 0.27) meta-analyses. With each outcome in each meta-analysis deleted from the model once, results remained statistically significant or trended towards statistical significance (*P* < 0.001 to 0.07) for both, ranging from an SMD of −0.31 to −0.50 in the Atlantis et al. meta-analysis [42] and −0.47 to −0.61 in the McGovern et al. meta-analysis [43].

No statistically significant changes in BMI or BMI-related outcomes were found for either the Atlantis et al. [42] (*P* = 0.11) or McGovern et al. [43] (*P* = 0.86) meta-analyses. In addition, overlapping confidence intervals were observed for both meta-analyses [42, 43]. For the McGovern et al. meta-analysis [43], no statistically significant heterogeneity or publication bias (*b*_0_, −0.62, 95% CI, −3.2 to 2.0, *P* = 0.60) was observed. With each outcome deleted from the model once, changes remained nonsignificant (*P* = 0.29 to 0.99), ranging from a SMD of −0.11 to 0.02 [43]. Insufficient BMI data were available to test for heterogeneity, publication bias, and influence analysis in the Atlantis et al. meta-analysis [42].

In addition to percent body fat and BMI, the Atlantis et al. meta-analysis also reported outcome results for body weight and central obesity (waist circumference and waist-to-hip ratio) [42]. A trend for statistically significant reductions in body weight was observed (*P* = 0.07) but 95% CIs were overlapping. Heterogeneity was found to be moderate and statistically significant. In addition, publication bias was also found to be statistically significant (*b*_0_, −1.7, 95% CI, −3.3 to −0.05, *P* = 0.05). With each outcome deleted from the model once, results were statistically significant, and all heterogeneity was removed when one outcome from one study was deleted from the model ($\overset{-}{X}\pm \text{SE}$, −3.7 ± 1.4 kg, 95% CI, −6.4 to −0.9, *P* = 0.009; *Q* = 7.0, *P* = 0.63, *I* ^2^ = 0%). For central obesity, a trend for statistical significance (*P* = 0.07) was reported but 95% CIs were overlapping. No statistically significant heterogeneity or publication bias (*b*_0_, −0.90, 95% CI, −4.7 to 2.9, *P* = 0.41) was observed. Results were in the direction of benefit (SMD, −0.19 to −0.29) but remained nonsignificant (*P* = 0.12 to 0.23) when each outcome was deleted from the model once.

#### 3.3.2. Other Analyses (Sensitivity, Subgroup, and Metaregression)

The Atlantis et al. meta-analysis conducted several additional analyses beyond the overall findings for percent body fat and body weight [42]. For percent body fat, reductions were greater (SMD, −0.6, 95% CI, −0.8 to −0.3, *P* < 0.001) when studies were limited to higher ($\overset{-}{X}\pm \text{SD}$, 177 ± 23 minutes per week) versus lower ($\overset{-}{X}\pm \text{SD}$, 153 ± 25 minutes per week, <3 days per week) doses of exercise as well as when strength training studies were removed from the model (SMD, −0.5, 95% CI, −0.8 to −0.1, *P* = 0.003). Results remained stable when separate analyses were conducted with studies that reported changes in dietary intake (*P* = 0.02) and exercise-only studies (*P* = 0.005) deleted from the models. Results were no longer statistically significant when studies that did not report exercise compliance or changes in exercise were deleted from the analysis (SMD, −0.3, 95% CI, −0.7 to 0.2, *P* = 0.11).

For changes in body weight, reductions were greater ($\overset{-}{X}$, −4.9 kg, 95% CI, −9.1 to −0.7, *P* = 0.01) when studies were limited to higher ($\overset{-}{X}\pm \text{SD}$, 156 ± 25 minutes per week) versus lower ($\overset{-}{X}\pm \text{SD}$, 117 ± 46 minutes per week, <3 days per week) doses of exercise as well as when strength training studies were removed from the model ($\overset{-}{X}$, −3.1 kg, 95% CI, −6.1 to −0.1, *P* = 0.02). With studies that reported dietary intake removed, reductions in body weight increased ($\overset{-}{X}$, −5.1 kg, 95% CI, −8.6 to −1.6, *P* = 0.002). In contrast, results were no longer statistically significant when studies that did not report exercise compliance or changes in exercise were deleted from the analysis ($\overset{-}{X}$, −2.3 kg, 95% CI, −6.8 to 2.1, *P* = 0.20).

For dose-response, no statistically significant associations were observed for changes in percent body fat, body weight, and central obesity when correlated with the volume of prescribed exercise (minutes per week) and total dose (minutes per week ∗ length of study in weeks) [42]. A trend was observed for greater reductions in body weight and exercise interventions that occurred over a greater number of weeks.

For the McGovern et al. meta-analysis [43], changes in percent body fat were reported to be greater than changes in BMI-related measures (*P* for interaction = 0.0007). However, when limited to trials that assessed both, results were no longer statistically significant (*P* = 0.28). No other analyses were reported from the McGovern et al. meta-analysis [43].

#### 3.3.3. NNT and Population Estimates

The NNT and gross estimates of the number of overweight and obese children and adolescents who might reduce their percent body fat from participation in an exercise program are shown in Table 3. As can be seen, the 95% CIs for the NNT and subsequent estimates from the Atlantis et al. meta-analysis [42] were wide, suggesting a lack of precision. Depending on the meta-analysis, approximately 2.8 to 3.6 million of the 12.5 million overweight and obese children in the US could reduce their percent body fat (ideally) by participating in a regular exercise program. Worldwide, an estimated 24.5 to 31.5 million might benefit.

## 4. Discussion

### 4.1. Findings

The purpose of the current study was to conduct a systematic review of previous meta-analyses addressing the effects of exercise (aerobic, strength training, or both) in the treatment of overweight and obesity in children and adolescents. Overall, it appears that exercise reduces percent body fat in overweight and obese children and adolescents. This interpretation is further supported by the robustness of results across both meta-analyses [42, 43] with respect to magnitude of effect, nonoverlapping confidence intervals, influence analysis (each study deleted from the model once) and publication bias. In contrast, heterogeneity and overlapping 95% PIs were found for the Atlantis et al. [42] but not McGovern et al. [43] meta-analysis. In addition, while the absolute NNT was similar across both meta-analyses [42, 43], the 95% CIs were wider for the Atlantis et al. study [42].

Given that the Atlantis et al. [42] study focused specifically on exercise, additional analyses were conducted. Most notably, greater reductions in percent body fat were found with higher exercise doses as well as when strength training studies were deleted from the model. While the former results appear plausible, the latter may be questioned. However, it is feasible that the potentially increased caloric expenditure from aerobic exercise may have resulted in greater reductions in percent body fat. The former notwithstanding, these results need to be interpreted with caution for at least two reasons. First, given the large number of statistical tests conducted, these findings could have been nothing more than the play of chance. Second, because studies are not randomly assigned to covariates, they are considered to be observational in nature [44]. Consequently, the results of moderator and regression analyses conducted in a meta-analysis do not support causal inferences. Nonetheless, these findings are probably important as they support the need for addressing these potential associations in future, well-designed, randomized controlled trials.

While improvements in percent body fat were observed, there is currently insufficient evidence that exercise improves BMI-related measures, body weight, and central obesity in overweight and obese children and adolescents. However, it is important to understand that a “lack of evidence of effect does not mean evidence of no effect” [45]. As additional evidence accumulates, one may gain a better understanding regarding the effects of exercise on these outcomes in overweight and obese children and adolescents.

### 4.2. Implications for Research

The results of the current systematic review of previous meta-analyses on the effects of exercise in the treatment of overweight and obese children and adolescents have several implications for future research. First, while the overall quality of the two meta-analyses was considered adequate, there are several areas that might be improved upon in future meta-analytic work. These include (1) avoiding the use of publication status as an inclusion criterion, (2) documenting and providing a list of not only included studies but also excluded studies, including the reasons for exclusion, and (3) assessing publication bias. The former notwithstanding, avoiding the use of publication status as an inclusion criterion could be questioned. For example, van Driel et al. [46] concluded that (1) the difficulty in retrieving unpublished work could lead to selection bias, (2) many unpublished trials are eventually published, (3) the methodological quality of such studies is poorer than those that are published, and (4) the effort and resources required to obtain unpublished work may not be warranted.

Second, both of the included studies were aggregate data meta-analyses [42, 43]. While this continues to be the most common type of meta-analysis, individual-participant data meta-analyses (IPD) are considered to be the gold standard when attempting to quantitatively combine data from different studies on the same topic [47]. Thus, future meta-analysts may want to consider using the IPD approach when addressing the effects of exercise in the treatment of overweight and obese children and adolescents. However, the use of the IPD approach needs to be considered with respect to the ability to retrieve IPD from investigators as well as the increased costs associated with the conduct of such [48], although methods to address the former have recently been developed [49].

Third, given the apparent lack of available data in the original studies included in the two meta-analyses [42, 43], there is a need for future randomized controlled trials to examine and report the safety and cost-effectiveness of their exercise intervention(s) in the treatment of overweight and obesity among children and adolescents. In addition, since the average length of studies in the included meta-analyses was only 16 [42] and 23 [43] weeks, a need exists for longer intervention studies, including follow-up studies, to more fully understand the longitudinal effects of exercise on adiposity. Similarly, the apparent focus on per-protocol versus intention-to-treat analyses in the original trials allows one to draw conclusions regarding the efficacy (does the treatment work?) but not the effectiveness (does the treatment work in the real world?) of exercise in the treatment of overweight and obese children and adolescents.

Fourth, the dose-response effects of exercise on measures of adiposity remain elusive. While the Atlantis et al. meta-analysis concluded that 155 to 180 minutes per week of moderate to high intensity exercise is effective for reducing body fat in overweight and obese children and adolescents [42], additional research on this topic is needed, especially with respect to body weight, BMI-related measures, and central obesity.

Fifth, percent body fat, but not BMI, appeared to be a more sensitive indicator of exercise-induced changes in adiposity among overweight and obese children and adolescents. Thus, future researchers may want to focus on percent body fat as their primary outcome despite the finding that BMI has been shown to correlate well with body fatness in children and adolescents [50]. However, while numerous methods exist for the assessment of percent body fat (skinfold calipers, hydrostatic weighing, whole body air-displacement plethysmography, dilution, dual energy X-ray absorptiometry, computerized tomography, magnetic resonance imaging) [45, 51], none may be practical in many settings, including the community-based setting. Thus, the use of BMI-related measures such as BMI *z*-score and BMI percentile may need to be considered but interpreted with the realization that they may not be very sensitive to change. In addition, given its simplicity, BMI is currently the universally accepted method for assessing adiposity in children and adolescents [52].

Sixth, because neither meta-analysis reported NNT [42, 43], it is suggested that future meta-analytic work includes such. From the investigative team's perspective, the reporting of such information is important because it provides practically relevant information to decision-makers (practitioners, policy-makers, etc.) regarding the effects of a selected treatment on an outcome. Along those lines, formulas now exist for calculating NNT from continuous data [53].

Finally, the most recent meta-analysis that met our inclusion criteria, published in 2008, included studies published up to February 2006, more than 7 years ago. While there is no definitive consensus regarding when to update a systematic review, with or without a meta-analysis, recent research by Pattanittum et al. [54] concluded that three practical statistical methods could be applied to examine the need to update systematic reviews, with or without meta-analysis. Such updated work is critical with respect to providing guidelines based on the most recent evidence available.

### 4.3. Implications for Practice

The results of the current systematic review provide several implications for practice. First, while there is a lack of cost-effectiveness and safety data, the use of exercise appears to be efficacious for improving adiposity, specifically percent body fat, in overweight and obese children and adolescents. The relatively low NNT observed as well as the potential number of overweight and obese children and adolescents who may benefit lends further support for this recommendation.

Second, while the dose-response effects of exercise in the treatment of overweight and obese children and adolescents have not been fully elucidated, it would appear prudent to recommend that practitioners follow the general recommendations for exercise in children and adolescents, that is, 60 minutes or more of physical activity each day [55]. The majority of the 60 minutes should be comprised of moderate to vigorous aerobic activity (bicycling, running, etc.) as well as muscle strengthening (pushups, etc.) and bone strengthening (jumping rope, etc.), 3 days per week [55]. Given the initial difficulty that overweight and obese children and adolescents may have in meeting these requirements, an individual exercise prescription that gradually progresses them to this level of effort seems appropriate.

Third, given the apparent lack of sensitivity of BMI, it is recommended that practitioners assess and track changes in adiposity using one of the numerous methods available for assessing percent body fat. If not possible, then the assessment of adiposity using BMI *z-*score or percentile can be used with the understanding that the true effects of exercise on adiposity in overweight and obese children and adolescents may not be fully realized with this approach.

Finally, given the observed magnitude of response of exercise on percent body fat in overweight and obese children and adolescents, exercise combined with other lifestyle and/or pharmacological interventions may be necessary for eliciting a health-improving impact on percent body fat in overweight and obese children and adolescents. Along those lines, an evaluation and treatment algorithm currently exists for addressing this issue [56].

### 4.4. Strengths and Potential Limitations of Current Study

There are several strengths to the current study. First, to the best of the authors' knowledge, this is the first systematic review of previous meta-analyses that has examined the effects of exercise in the treatment of overweight and obese adolescents, an increasingly important approach for addressing the effects of various healthcare interventions [12]. Second, the additional analyses conducted based on the available data (influence analysis, publication bias, etc.) helped strengthen the validity and findings of the two included meta-analyses [42, 43]. For example, the finding of no apparent publication bias for those studies that examined changes in percent body fat helped to strengthen the conclusions regarding the effects of exercise on percent body fat in overweight and obese children and adolescents. While conducting additional analyses beyond those reported in an original meta-analysis does not appear to be common when conducting systematic reviews of previous meta-analyses, future investigators may want to incorporate this methodology into their reviews while at the same time considering the additional time and effort involved in such an endeavor. Third, the NNT and gross estimates of the absolute number of overweight and obese children and adolescents who might reduce their percent body fat by participating in a regular exercise program were provided. In the authors' opinion, such estimates enhance the applicability and importance of findings. Fourth, the calculation and inclusion of PIs for statistically significant outcomes in the current study provide investigators with information that can aid them in planning future randomized controlled trials.

In addition to the strengths of the current study, there are several potential limitations. First, the investigative team established fairly strict eligibility criteria for the current systematic review. As a result, only two previous meta-analyses met all eligibility criteria [42, 43]. While more focused and applicable, other relevant issues such as the effects of exercise on quality-of-life in overweight and obese children and adolescents were not captured. Second, the gross population estimates for the number of children who could reduce their percent body fat by participating in an exercise program assumed that none of the overweight and obese children and adolescents were exercising regularly. Consequently, these numbers might be inflated. Finally, as with any systematic review, many of the biases inherent in both the included meta-analyses as well as the original trials that comprise each meta-analysis may also be present in a systematic review of previous meta-analyses.

## 5. Conclusions

The results of the current systematic review of previous meta-analyses suggest that exercise is efficacious for reducing percent body fat in overweight and obese children and adolescents. However, there is currently insufficient evidence to suggest that exercise reduces BMI-related measures, body weight, and central obesity in overweight and obese children and adolescents.

## Supplementary Material

Supplementary Material 1. Search strategies for databases searched. This supplementary file provides a description of the search strategy used for each database searched.
Supplementary Material 2. Studies excluded, including reasons for exclusion. This supplementary file provides a list of excluded studies, including the reasons for exclusion.
Supplementary Material 3. Item by item results using the AMSTAR assessment instrument. This supplementary file provides item by item results of methodological quality using the AMSTAR assessment instrument.

## Figures and Tables

**Table 1:** General characteristics of included meta-analyses.

Meta-analyses	Studies	Participants	Interventions	Outcomes
Atlantis et al. [42]	14	481 boys and girls 8–16 years of age ($\overset{-}{X}\pm \text{SD}$, 10.9 ± 1.5)	Supervised/unsupervised aerobics, strength training, aerobics + strength training; length ($\overset{-}{X}\pm \text{SD}$, 16 ± 7 weeks), frequency (2–5x week), duration (10–60 minutes per session), intensity (aerobics 60%–80% VO_2max⁡_; strength 50%–100% 10RM), compliance (51%–88%), minutes per week (120–180)	Body weight, BMI, percent body fat using DEXA, bioimpedence and hydrodensitometry, central obesity (waist circumference, waist-to-hip ratio, visceral adipose tissue)

McGovern et al. [43]	17	791 boys and girls (410 exercise, 381 control), approximately 5–18 years of age ($\overset{-}{X}\pm \text{SD}$, 11.9 ± 2.1)	Supervised/unsupervised aerobics, strength training, aerobics + strength training; length (6–104 weeks, $\overset{-}{X}\pm \text{SD}$, 23 ± 23), frequency (1–7x week, $\overset{-}{X}\pm \text{SD}$, 4 ± 1), duration (30–75 minutes per session, $\overset{-}{X}\pm \text{SD}$ = 53 ± 15), 90–200 minutes per week $(\overset{-}{X}\pm \text{SD}$ = 157 ± 34)	BMI + (kg/m^2^, *z*-score, percentile, percent overweight, adjusted for height), percent body fat

Notes: $\overset{-}{X}\pm \text{SD}$: mean ± standard deviation; VO_2max⁡_: maximum oxygen consumption; BMI: body mass index; DEXA: dual-energy X-ray absorptiometry.

**Table 2:** Overall findings of included meta-analyses.

Meta-analyses	ES/participants (No.)	Mean (95% CI)	*Q* (*p*)	*I* ^2^ (%)	PI (95%)
Atlantis et al. [42]					
(i) Percent fat (SMD)	9/369	**−0.4 (−0.7, −0.1)**	15.5 (0.05)*	48.4	−1.3, 0.5
(ii) BMI	3/71	−1.2 (−3.1, 0.8)	—	—	NA
(iii) Body weight (kg)	11/334	−2.7 (−6.1, 0.8)	17.9 (0.07)	44.1	NA
(iv) Central obesity (SMD)	4/156	−0.2 (−0.5, 0.06)	1.4 (0.70)	0	NA
McGovern et al. [43]					
(i) Percent fat (SMD)	6/358	**−0.5 (−0.7, −0.3)**	2.2 (0.81)	0	**−0.8, −0.2**
(ii) BMI-related (SMD)	11/433	−0.02 (−0.21, 0.18)	9.4 (0.49)	0	NA

Notes: No: Number; ES: effect size; CI: confidence intervals; *Q*: Cochran's *Q* statistic and associated alpha (*p*) value for heterogeneity; *I* ^2^: *I*-squared statistic for heterogeneity; PI: prediction intervals, based on a random effects model; SMD: standardized mean difference; central obesity measures derived from waist circumference, waist-to-hip ratio, visceral adipose tissue; BMI-related measures include body mass index (BMI) in kg/m^2^, BMI *z*-score, BMI percentile, and percent overweight, adjusted for height. NA: not applicable; —: insufficient data to calculate; mean (95% CI) based on random effects model; boldfaced values indicate continuous data with non-overlapping confidence intervals; *statistically significant, *P* ≤ 0.05.

**Table 3:** NNT and estimates of effect, in millions, for percent body fat.

Study	NNT (95% CI)	E1 (95% CI) (millions)	E2 (95% CI) (millions)
Atlantis et al. [42]	4 (3, 18)	2.8 (4.7, 0.7)	24.5 (41.7, 0.6)
McGovern et al. [43]	3 (3, 6)	3.6 (4.7, 2.1)	31.5 (41.7, 18.5)

Notes: NNT: number-needed-to treat; E1: estimate 1 derived from number of obese children and adolescents in the United States who could benefit from exercise (based on a previously estimated 12.5 million obese children and adolescents) [4]; E2: estimate 2 derived from number of overweight and obese children in the world who could benefit from exercise (based on a previously estimated 110 million overweight or obese children) [2, 38].
