# Supplementary material for: Effects of Exercise in the Treatment of Overweight and Obese Children and Adolescents: A Systematic Review of Meta-Analyses
Source: J Obes. 2013 Dec 24;2013:783103. doi: 10.1155/2013/783103 (PMC3886589; doi:10.1155/2013/783103)
Supplement: Supplementary file 1 — Supplementary Material 1. Search strategies for databases searched. This supplementary file provides a description of the search strategy used for each database searched. Supplementary Material 2. Studies excluded, including reasons for exclusion. This supplementary file provides a list of excluded studies, including the reasons for exclusion. Supplementary Material 3. Item by item results using the AMSTAR assessment instrument. This supplementary file provides item by item results of methodological quality using the AMSTAR assessment instrument. [file 783103.f1.docx]

**Supplementary File 1.** Search strategies for databases searched.
