# Supplementary material for: Effects of Exercise in the Treatment of Overweight and Obese Children and Adolescents: A Systematic Review of Meta-Analyses
Source: J Obes. 2013 Dec 24;2013:783103. doi: 10.1155/2013/783103 (PMC3886589; doi:10.1155/2013/783103)
Supplement: Supplementary file 2 [file 783103.f2.docx]

**Supplementary File 2.** Studies excluded, including reasons for exclusion.

(1) Systematic review of interventions in the treatment and prevention of obesity (Structured abstract). Database of Abstracts of Reviews of Effects 1997;149. Inappropriate Study Design

(2) ABSTRACTS. Journal of Orthopaedic & Sports Physical Therapy 2004 February;34(2):86-94. Inappropriate Study Design

(3) Behavioural interventions for type 2 diabetes: an evidence-based analysis. Ont Health Technol Assess Ser 2009;9(21):1-45. Inappropriate Study Design, Inappropriate Population

(4) Continuous Subcutaneous Insulin Infusion (CSII) Pumps for Type 1 and Type 2 Adult Diabetic Populations: An Evidence-Based Analysis. Ont Health Technol Assess Ser 2009;9(20):1-58. Inappropriate Study Design, Inappropriate Population

(5) Thin and Happy, or Fat and Sad: Are They Related? Journal of Sport & Exercise Psychology 2009 February;31(1):123. Inappropriate Study Design

(6) Design of a family-based lifestyle intervention for youth with type 2 diabetes: the TODAY study. Int J Obes (Lond) 2010 February;34(2):217-26. Inappropriate Study Design

(7) The Joanna Briggs Institute Best Practice Information Sheet: The effectiveness of pelvic floor muscle exercises on urinary incontinence in women following childbirth. Nursing & Health Sciences 2011;13(3):378-81. Inappropriate Study Design

(8) National Student Conference of the Canadian Society for Epidemiology and Biostatistics, Saskatoon, Canada, May 13-14, 2012 Abstracts. American Journal of Epidemiology 2012;176(1):80. Inappropriate Population, Inappropriate Study Design

(9) Adeniyi FB, Young T. Weight loss interventions for chronic asthma. Cochrane Database of Systematic Reviews 2012;(7). Inappropriate Study Design

(10) Agras WS, Mascola AJ. Risk factors for childhood overweight. Current Opinion in Pediatrics 2005;17(5):648-52. Inappropriate Study Design

(11) Ahn S, Fedewa AL. A Meta-analysis of the Relationship Between Children's Physical Activity and Mental Health. Journal of Pediatric Psychology 2011;36(4):385-97. Inappropriate Outcomes

(12) Allison DB, Faith MS, Gorman BS. Publication bias in obesity treatment trials? International Journal of Obesity 1996;20(10):931-7. Inappropriate Study Design

(13) Amorim AR, Linne YM, Lourenco PM. Diet or exercise, or both, for weight reduction in women after childbirth. Cochrane Database Syst Rev 2007 July 18;(3):CD005627. Inappropriate Population

(14) An JY, Hayman LL, Park YS, Dusaj TK, Ayres CG. Web-Based Weight Management Programs for Children and Adolescents A Systematic Review of Randomized Controlled Trial Studies. Advances in Nursing Science 2009;32(3):222-40. Inappropriate Study Design, Inappropriate Intervention

(15) Anderson JW, Konz EC, Frederich RC, Wood CL. Long-term weight-loss maintenance: a meta-analysis of US studies. American Journal of Clinical Nutrition 2001;74(5):579-84. Inappropriate Population, Inappropriate Intervention

(16) Armstrong N, Simons-Morton B. Physical activity and blood lipids in adolescents. Pediatric Exercise Science 1994;6(4):381-405. Inappropriate Study Design, Inappropriate Outcomes

(17) Askie LM, Baur LA, Campbell K, Daniels LA, Hesketh K, Magarey A, Mihrshahi S, Rissel C, Simes J, Taylor B, Taylor R, Voysey M, Wen LM. The Early Prevention of Obesity in CHildren (EPOCH) Collaboration - an Individual Patient Data Prospective Meta-Analysis. Bmc Public Health 2010;10. Inappropriate Study Design

(18) Atkin AJ, Gorely T, Biddle SJ, Cavill N, Foster C. Interventions to promote physical activity in young people conducted in the hours immediately after school: a systematic review. International Journal of Behavioral Medicine 2011;18(3):176-87. Inappropriate Study Design, Inappropriate Outcomes

(19) Barba M, Schunemann HJ, Sperati F, Akl EA, Musicco F, Guyatt G, Muti p. The effects of metformin on endogenous androgens and SHBG in women: a systematic review and meta-analysis. Clinical Endocrinology 2009;70(5):661-70. Inappropriate Population, Inappropriate Intervention

(20) Barr-Anderson DJ, Adams-Wynn AW, DiSantis KI, Kumanyika S. Family-focused physical activity, diet and obesity interventions in African-American girls: a systematic review. Obesity Reviews 2013;14(1):29-51. Inappropriate Study Design, Inappropriate Outcomes

(21) Barshop NJ, Sirlin CB, Schwimmer JB, Lavine JE. Review article: epidemiology, pathogenesis and potential treatments of paediatric non-alcoholic fatty liver disease. Aliment Pharmacol Ther 2008 July;28(1):13-24. Inappropriate Study Design

(22) Bautista-Castana I, Doreste J, Serra-Majem L. Effectiveness of interventions in the prevention of childhood obesity. European Journal of Epidemiology 2004;19(7):617-22. Inappropriate Study Design

(23) Beets MW, Beighle A, Erwin HE, Huberty JL. After-School Program Impact on Physical Activity and Fitness A Meta-Analysis. American Journal of Preventive Medicine 2009;36(6):527-37. Inappropriate Study Design, Inappropriate Population

(24) Beets MW, Bornstein D, Beighle A, Cardinal BJ, Morgan CF. Pedometer-Measured Physical Activity Patterns of Youth A 13-Country Review. American Journal of Preventive Medicine 2010;38(2):208-16. Inappropriate Outcomes, Inappropriate Study Design

(25) Beets MW, Tilley F, Kim Y, Webster C. Nutritional policies and standards for snacks served in after-school programmes: a review. Public Health Nutrition 2011;14(10):1882-90. Inappropriate Outcomes, Inappropriate Study Design

(26) Benson AC, Torode ME, Singh MAF. Effects of resistance training on metabolic fitness in children and adolescents: a systematic review. Obesity Reviews 2008;9(1):43-66. Inappropriate Study Design, Inappropriate Population

(27) Berry D, Sheehan R, Heschel R, Knafl K, Melkus G, Grey M. Family-based interventions for childhood obesity: a review. Journal of Family Nursing 2004;10(4):429-49. Inappropriate Study Design, Inappropriate Intervention

(28) Blohm D, Ploch T, Apelt S. Efficacy of exercise therapy to reduce cardiometabolic risk factors in overweight and obese children and adolescents: a systematic review. Deutsche Medizinische Wochenschrift 2012;137(50):2631-6. Inappropriate Study Design

(29) Bond M, Wyatt K, Lloyd J, Welch K, Taylor R. Systematic review of the effectiveness and cost-effectiveness of weight management schemes for the under fives: a short report. Health Technology Assessment 2009;13(61):1-+. Inappropriate Study Design, Inappropriate Outcomes

(30) Bond M, Wyatt K, Lloyd J, Taylor R. Systematic review of the effectiveness of weight management schemes for the under fives. Obesity Reviews 2011;12(4):242-53. Inappropriate Study Design

(31) Brandt S, Moss A, Berg S, Wabitsch M. School-based obesity prevention. Bundesgesundheitsblatt-Gesundheitsforschung-Gesundheitsschutz 2010;53(2-3):207-20. Inappropriate Study Design

(32) Branscum p, Sharma M. A systematic analysis of childhood obesity prevention interventions targeting Hispanic children: lessons learned from the previous decade. Obesity Reviews 2011;12(501):e151-e158. Inappropriate Study Design

(33) Brockow T, Conradi E, Ebenbichler G, Michalsen A, Resch KL. The Role of Mild Systemic Heat and Physical Activity on Endothelial Function in Patients with Increased Cardiovascular Risk: Results from a Systematic Review. Forschende Komplementarmedizin 2011;18(1):24-30. Inappropriate Population

(34) Brosseau L, Wells GA, Tugwell p, Egan M, Dubouloz CJ, Casimiro L, Bugnariu N, Welch VA, De Angelis G, Francoeur L, Milne S, Loew L, McEwan J, Messier SP, Doucet E, Kenny GP, Prud'homme D, Lineker S, Bell M, Poitras Sp. Ottawa Panel Evidence-Based Clinical Practice Guidelines for the Management of Osteoarthritis in Adults Who Are Obese or Overweight. Physical Therapy 2011 June;91(6):843-61. Inappropriate Study Design, Inappropriate Population

(35) Brown HE, Pearson N, Braithwaite RE, Brown WJ, Biddle SJ. Physical activity interventions and depression in children and adolescents : a systematic review and meta-analysis. Sports Med 2013 March;43(3):195-206. Inappropriate Outcomes, Inappropriate Population

(36) Brown HS, Perez A, Li YP, Hoelscher DM, Kelder SH, Rivera R. The cost-effectiveness of a school-based overweight program. International Journal of Behavioral Nutrition and Physical Activity 2007;4:47. Inappropriate Study Design, Inappropriate Outcomes

(37) Brown MJ, Sinclair M, Liddle D, Hill AJ, Madden E, Stockdale J. A Systematic Review Investigating Healthy Lifestyle Interventions Incorporating Goal Setting Strategies for Preventing Excess Gestational Weight Gain. Plos One 2012;7(7). Inappropriate Study Design

(38) Brown RJ, de Banate MA, Rother KI. Artificial Sweeteners: A systematic review of metabolic effects in youth. International Journal of Pediatric Obesity 2010;5(4):305-12. Inappropriate Intervention, Inappropriate Study Design

(39) Brown T, Avenell A, Edmunds LD, Moore H, Whittaker V, Avery L, Summerbell C. Systematic review of long-term lifestyle interventions to prevent weight gain and morbidity in adults. Obes Rev 2009 November;10(6):627-38. Inappropriate Study Design, Inappropriate Population

(40) Brown T, Summerbell C. Systematic review of school-based interventions that focus on changing dietary intake and physical activity levels to prevent childhood obesity: an update to the obesity guidance produced by the National Institute for Health and Clinical Excellence. Obesity Reviews 2009;10(1):110-41. Inappropriate Study Design

(41) Cain N, Sitz L, Kutz M. The occupational therapist's role in the treatment of child and adolescent overweight and obesity: A systematic literature review. United States -- Minnesota: The College of St. Scholastica; 2010. Inappropriate Study Design

(42) Campbell F, Johnson M, Messina J, Guillaume L, Goyder E. Behavioural interventions for weight management in pregnancy: A systematic review of quantitative and qualitative data. Bmc Public Health 2011;11. Inappropriate Population

(43) Campbell K, Waters E, O'Meara S, Summerbell C. Interventions for preventing obesity in childhood. A systematic review. Obes Rev 2001 August;2(3):149-57. Inappropriate Study Design

(44) Campbell KJ, Hesketh KD. Strategies which aim to positively impact on weight, physical activity, diet and sedentary behaviours in children from zero to five years. A systematic review of the literature. Obesity Reviews 2007;8(4):327-38. Inappropriate Study Design, Inappropriate Outcomes

(45) Carter FA, Bulik CM. Childhood obesity prevention programs: how do they affect eating pathology and other psychological measures? Psychosom Med 2008 April;70(3):363-71. Inappropriate Study Design, Inappropriate Outcomes

(46) Choi J, Joseph L, Pilote L. Obesity and C-reactive protein in various populations: a systematic review and meta-analysis. Obesity Reviews 2013;14(3):232-44. Inappropriate Study Design, Inappropriate Comparison Group

(47) Ciampa PJ, Kumar D, Barkin SL, Sanders LM, Yin HS, Perrin EM, Rothman RL. Interventions aimed at decreasing obesity in children younger than 2 years: a systematic review. Archives of Pediatrics and Adolescent Medicine 2010;164(12):1098-104. Inappropriate Study Design, Inappropriate Population

(48) Cislak A, Safron M, Pratt M, Gaspar T, Luszczynska A. Family-related predictors of body weight and weight-related behaviours among children and adolescents: a systematic umbrella review. Child Care Health and Development 2012;38(3):321-31. Inappropriate Study Design

(49) Clemmens D, Hayman LL. Increasing activity to reduce obesity in adolescent girls: A research review. Jognn-Journal of Obstetric Gynecologic and Neonatal Nursing 2004;33(6):801-8. Inappropriate Study Design

(50) Cliff DP, Okely AD, Morgan PJ, Jones RA, Steele JR. The impact of child and adolescent obesity treatment interventions on physical activity: a systematic review. Obesity Reviews 2010;11(7):516-30. Inappropriate Study Design

(51) Cole-Lewis H, Kershaw T. Text messaging as a tool for behavior change in disease prevention and management. Epidemiologic Reviews 2010;32(1):56-69. Inappropriate Study Design, Inappropriate Intervention

(52) Collins CE, Warren J, Neve M, McCoy p, Stokes BJ. Measuring effectiveness of dietetic interventions in child obesity: A systematic review of randomized trials. Archives of Pediatrics & Adolescent Medicine 2006 September 1;160(9):906-22. Inappropriate Intervention

(53) Collins CE, Warren JM, Neve M, McCoy p, Stokes B. Systematic review of interventions in the management of overweight and obese children which include a dietary component. International Journal of Evidence-Based Healthcare 2007;5:2-53. Inappropriate Intervention

(54) Connelly JB, Uaso MJ, Butler G. A systematic review of controlled trials of interventions to prevent childhood obesity and overweight: A realistic synthesis of the evidence. Public Health 2007;121(7):510-7. Inappropriate Study Design

(55) Cook-Cottone C, Casey CM, Feeley TH, Baran J. A Meta-Analytic Review of Obesity Prevention in the Schools: 1997-2008. Psychology in the Schools 2009;46(8):695-719. Inappropriate Study Design, Inappropriate Comparison Group

(56) Coutant R, Carel JC, Timsit J, Boitard C, Bougneres p. Insulin and the prevention of insulin-dependent diabetes mellitus. Diabetes Metab 1997 September;23 Suppl 3:25-8.:25-8. Inappropriate Study Design, Inappropriate Outcomes

(57) Cuijpers p, van SA, Andersson G. Internet-administered cognitive behavior therapy for health problems: a systematic review. Journal of Behavioral Medicine 2008;31(2):169-77. Inappropriate Intervention, Inappropriate Population

(58) Cutler JA. Randomized clinical trials of weight reduction in nonhypertensive persons. Ann Epidemiol 1991 May;1(4):363-70. Inappropriate Study Design

(59) da Silveira JAC, Taddei JADC, Guerra PH, Nobre MRC. The effect of participation in school-based nutrition education interventions on body mass index: A meta-analysis of randomized controlled community trials. Preventive Medicine 2013;56(3-4):237-43. Inappropriate Intervention

(60) Dalton WT, III, Schetzina KE, Holt N, Fulton-Robinson H, Ho AL, Tudiver F, McBee MT, Wu T. Parent-Led Activity and Nutrition (PLAN) for healthy living: design and methods. Contemp Clin Trials 2011 November;32(6):882-92. Inappropriate Study Design

(61) Damiano DL, DeJong SL. A systematic review of the effectiveness of treadmill training and body weight support in pediatric rehabilitation. Journal of Neurologic Physical Therapy 2009;33(1):27-44. Inappropriate Population

(62) Davis JN, Ventura EE, Shaibi GQ, Byrd-Williams CE, Alexander KE, Vanni AK, Meija MR, Weigensberg MJ, Spruijt-Metz D, Goran MI. Interventions for improving metabolic risk in overweight Latino youth. Int J Pediatr Obes 2010 October;5(5):451-5. Inappropriate Study Design

(63) De Bourdeaudhuij I, Van Cauwenberghe E, Spittaels H, Oppert JM, Rostami C, Brug J, Van Lenthe F, Lobstein T, Maes L. School-based interventions promoting both physical activity and healthy eating in Europe: a systematic review within the HOPE project. Obesity Reviews 2011;12(3):205-16. Inappropriate Study Design

(64) Decsi T, Molnar D. Insulin resistance syndrome in children : pathophysiology and potential management strategies. Paediatr Drugs 2003;5(5):291-9. Inappropriate Study Design, Inappropriate Outcomes

(65) DeMattia L, Lemont L, Meurer L. Do interventions to limit sedentary behaviours change behaviour and reduce childhood obesity: a critical review of the literature. Obesity Reviews 2007;8(1):69-81. Inappropriate Study Design

(66) Demetriou Y, Honer O. Physical activity interventions in the school setting: A systematic review. Psychology of Sport and Exercise 2012;13(2):186-96. Inappropriate Study Design

(67) Dietz p, Hoffmann S, Lachtermann E, Simon p. Influence of Exclusive Resistance Training on Body Composition and Cardiovascular Risk Factors in Overweight or Obese Children: A Systematic Review. Obesity Facts 2012;5(4):546-60. Inappropriate Study Design

(68) Doak CM, Visscher TL, Renders CM, Seidell JC. The prevention of overweight and obesity in children and adolescents: a review of interventions and programmes. Obesity Reviews 2006;7(1):111-36. Inappropriate Study Design

(69) Dobbins M, Lockett D, Michel I, Beyers J, Feldman L, Vohra J. The effectiveness of school-based interventions in promoting physical activity and fitness among children and youth: A systematic review (Report). Hamilton, ON: Effective Public Health Practice Project (EPHPP); 2001 Jan 1. Inappropriate Study Design

(70) Dobbins M, Husson H, DeCorby K, LaRocca RL. School-based physical activity programs for promoting physical activity and fitness in children and adolescents aged 6 to 18. Cochrane Database of Systematic Reviews 2013;(2). Inappropriate Outcomes

(71) Dodd JM, Grivell RM, Crowther CA, Robinson JS. Antenatal interventions for overweight or obese pregnant women: a systematic review of randomised trials. BJOG An International Journal of Obstetrics and Gynaecology 2010;117(11):1316-26. Inappropriate Population

(72) Douketis JD, Macie C, Thabane L, Williamson DF. Systematic review of long-term weight loss studies in obese adults: clinical significance and applicability to clinical practice. International Journal of Obesity 2005;29(10):1153-67. Inappropriate Population

(73) Dratva J, Real FG, Schindler C, Ackermann-Liebrich U, Gerbase MW, Probst-Hensch NM, Svanes C, Omenaas ER, Neukirch F, Wjst M, Morabia A, Jarvis D, Leynaert B, Zemp E. Is age at menopause increasing across Europe? Results on age at menopause and determinants from two population-based studies. Menopause-the Journal of the North American Menopause Society 2009;16(2):385-94. Inappropriate Study Design, Inappropriate Population

(74) Dunn AL, Andersen RE, Jakicic JM. Lifestyle physical activity interventions. History, short- and long-term effects, and recommendations. Am J Prev Med 1998 November;15(4):398-412. Inappropriate Study Design, Inappropriate Outcomes

(75) Eakin EG, Bull SS, Glasgow RE, Mason M. Reaching those most in need: a review of diabetes self-management interventions in disadvantaged populations. Diabetes/Metabolism Research and Reviews 2002;18(1):26-35. Inappropriate Population

(76) Eksambi SH. Strategies for obesity prevention in child care settings: A literature review. United States -- Texas: The University of Texas School of Public Health; 2010. Inappropriate Study Design

(77) Ells LJ, Hillier FC, Shucksmith J, Crawley H, Harbige L, Shield J, Wiggins A, Summerbell CD. A systematic review of the effect of dietary exposure that could be achieved through normal dietary intake on learning and performance of school-aged children of relevance to UK schools. Br J Nutr 2008 November;100(5):927-36. Inappropriate Study Design, Inappropriate Outcomes

(78) Epstein LH, Coleman KJ, Myers MD. Exercise in treating obesity in children and adolescents. Med Sci Sports Exerc 1996 April;28(4):428-35. Inappropriate Study Design

(79) Epstein LH, Myers MD, Raynor HA, Saelens BE. Treatment of pediatric obesity. Pediatrics 1998 March;101(3 Pt 2):554-70. Inappropriate Study Design

(80) Epstein LH, Goldfield GS. Physical activity in the treatment of childhood overweight and obesity: current evidence and research issues. Medicine and Science in Sports and Exercise 1999 November;31(11 Suppl):S553-S559. Inappropriate Study Design

(81) Escalante Y, Saavedra JM, Garcia-Hermoso A, Dominguez AM. Improvement of the lipid profile with exercise in obese children: A systematic review. Preventive Medicine 2012;54(5):293-301. Inappropriate Outcomes

(82) Fabricatore AN, Wadden TA, Higginbotham AJ, Faulconbridge LF, Nguyen AM, Heymsfield SB, Faith MS. Intentional weight loss and changes in symptoms of depression: a systematic review and meta-analysis. International Journal of Obesity 2011;35(11):1363-76. Inappropriate Outcomes

(83) Faith MS, Van HL, Appel LJ, Burke LE, Carson JA, Franch HA, Jakicic JM, Kral TV, Odoms-Young A, Wansink B, Wylie-Rosett J. Evaluating parents and adult caregivers as "agents of change" for treating obese children: evidence for parent behavior change strategies and research gaps: a scientific statement from the American Heart Association. Circulation 2012 March 6;125(9):1186-207. Inappropriate Study Design

(84) Festi D, Colecchia A, Sacco T, Bondi M, Roda E, Marchesini G. Hepatic steatosis in obese patients: clinical aspects and prognostic significance. Obesity Reviews 2004 February;5(1):27-42. Inappropriate Study Design

(85) Flodmark CE, Marcus C, Britton M. Interventions to prevent obesity in children and adolescents: a systematic literature review. International Journal of Obesity 2006;30(4):579-89. Inappropriate Study Design

(86) Flynn MAT, Mcneil DA, Maloff B, Mutasingwa D, Wu M, Ford C, Tough SC. Reducing obesity and related chronic disease risk in children and youth: a synthesis of evidence with 'best practice' recommendations. Obesity Reviews 2006;7:7-66. Inappropriate Study Design

(87) Foltz JL, May AL, Belay B, Nihiser AJ, Dooyema CA, Blanck HM. Population-Level Intervention Strategies and Examples for Obesity Prevention in Children. Annual Review of Nutrition, Vol 32 2012;32:391-+. Inappropriate Study Design

(88) Friedemann C, Heneghan C, Mahtani K, Thompson M, Perera R, Ward AM. Cardiovascular disease risk in healthy children and its association with body mass index: systematic review and meta-analysis. British Medical Journal 2012;345. Inappropriate Comparison Group

(89) Friedrich RR, Schuch I, Wagner MB. Effect of interventions on the body mass index of school-age students. Revista de Saude Publica 2012;46(3):551-60. Inappropriate Population

(90) Ganann R, Fitzpatrick-Lewis D, Ciliska D, Peirson L. Community-based interventions for enhancing access to or consumption of fruit and vegetables among five to 18-year olds: a scoping review. Bmc Public Health 2012;12. Inappropriate Study Design, Inappropriate Outcomes

(91) Gardner B, Wardle J, Poston L, Croker H. Changing diet and physical activity to reduce gestational weight gain: a meta-analysis. Obesity Reviews 2011;12(7):E602-E620. Inappropriate Population

(92) Geleijnse JM, Kok FJ, Grobbee DE. Impact of dietary and lifestyle factors on the prevalence of hypertension in Western populations. European Journal of Public Health 2004 September;14(3):235-9. Inappropriate Population, Inappropriate Outcomes

(93) Gerards SMPL, Sleddens EFC, Dagnelie PC, De Vries NK, Kremers SPJ. Interventions addressing general parenting to prevent or treat childhood obesity. International Journal of Pediatric Obesity 2011 January 1;6(2 -2):e28-e45. Inappropriate Study Design, Inappropriate Intervention

(94) Gibson LJ, Peto J, Warren JM, dos SS, I. Lack of evidence on diets for obesity for children: a systematic review. International Journal of Epidemiology 2006;35(6):1544-52. Inappropriate Intervention

(95) Gillett M, Royle p, Snaith A, Scotland G, Poobalan A, Imamura M, Black C, Boroujerdi M, Jick S, Wyness L, McNamee p, Brennan A, Waugh N. Non-pharmacological interventions to reduce the risk of diabetes in people with impaired glucose regulation: a systematic review and economic evaluation. Health Technology Assessment 2012 August;16(33):1-iv. Inappropriate Study Design, Inappropriate Outcomes

(96) Glenny AM, O'Meara S, Melville A, Sheldon TA, Wilson C. The treatment and prevention of obesity: a systematic review of the literature. International Journal of Obesity and Related Metabolic Disorders 1997 September;21(9):715-37. Inappropriate Study Design

(97) Goldfield GS, Epstein LH, Kilanowski CK, Paluch RA, Kogut-Bossler B. Cost-effectiveness of group and mixed family-based treatment for childhood obesity. International Journal of Obesity 2001;25(12):1843-9. Inappropriate Intervention, Inappropriate Comparison Group

(98) Goldfield GS, Harvey A, Grattan K, Adamo KB. Physical activity promotion in the preschool years: a critical period to intervene. Int J Environ Res Public Health 2012 April;9(4):1326-42. Inappropriate Study Design

(99) Gonzalez-Suarez C, Worley A, Grimmer-Somers K, Dones V. School-Based Interventions on Childhood Obesity A Meta-Analysis. American Journal of Preventive Medicine 2009;37(5):418-27. Inappropriate Outcomes

(100) Gourlan MJ, Trouilloud DO, Sarrazin PG. Interventions promoting physical activity among obese populations: a meta-analysis considering global effect, long-term maintenance, physical activity indicators and dose characteristics. Obesity Reviews 2011;12(7):E633-E645. Inappropriate Outcomes

(101) Guelinckx I, Devlieger R, Vansant G. Pregnancies complicated by obesity: clinical approach and nutritional management. Verh K Acad Geneeskd Belg 2010;72(5-6):253-76. Inappropriate Study Design, Inappropriate Population

(102) Guillaume M, Lapidus L, Beckers F, Lambert A, Bjorntorp p. Familial trends of obesity through three generations: the Belgian-Luxembourg child study. International Journal of Obesity and Related Metabolic Disorders 1995 September;19 Suppl 3:S5-9.:S5-S9. Inappropriate Study Design, Inappropriate Comparison Group

(103) Guinhouya BC, Samouda H, Zitouni D, Vilhelm C, Hubert H. Evidence of the influence of physical activity on the metabolic syndrome and/or on insulin resistance in pediatric populations: a systematic review. International Journal of Pediatric Obesity 2011;6(5-6):361-88. Inappropriate Outcomes, Inappropriate Population

(104) Hamel LM, Robbins LB, Wilbur J. Computer- and web-based interventions to increase preadolescent and adolescent physical activity: a systematic review. Journal of Advanced Nursing 2011;67(2):251-68. Inappropriate Intervention, Inappropriate Study Design

(105) Hamel LM, Robbins LB. Computer- and web-based interventions to promote healthy eating among children and adolescents: a systematic review. Journal of Advanced Nursing 2013;69(1):16-30. Inappropriate Intervention

(106) Han S, Middleton p, Crowther CA. Exercise for pregnant women for preventing gestational diabetes mellitus. Cochrane Database of Systematic Reviews: Reviews 2012;Issue 7. Inappropriate Population

(107) Haney EM, Hoyt HL, Bougatsos C, Freeman M, Fu R, Steiner RD, Helfand M, Nelson HD. Screening for lipid disorders in children and adolescents: systematic evidence review for the U.S. Preventive Services Task Force. Report. 2007. Inappropriate Intervention, Inappropriate Outcomes

(108) Haney EM, Huffman LH, Bougatsos C, Freeman M, Fu R, Steiner RD, Helfand M, Nelson HD, Kitzman-Ulrich H, Wilson DK, St George SM, Lawman H, Segal M, Fairchild A. The integration of a family systems approach for understanding youth obesity, physical activity, and dietary programs. Clinical Child and Family Psychology Review 2010 September;13(3):231-53. Inappropriate Population, Inappropriate Outcomes

(109) Hardeman W, Griffin S, Johnston M, Kinmonth AL, Wareham NJ. Interventions to prevent weight gain: a systematic review of psychological models and behaviour change methods. International Journal of Obesity 2000;24(2):131-43. Inappropriate Study Design

(110) Harris KC, Kuramoto LK, Schulzer M, Retallack JE. Effect of school-based physical activity interventions on body mass index in children: a meta-analysis. Canadian Medical Association Journal 2009;180(7):719-26. Inappropriate Population

(111) Harriss DJ, Atkinson G, George K, Cable NT, Reilly T, Haboubi N, Zwahlen M, Egger M, Renehan AG. Lifestyle factors and colorectal cancer risk (1): systematic review and meta-analysis of associations with body mass index. Colorectal Dis 2009 July;11(6):547-63. Inappropriate Population, Inappropriate Outcomes

(112) Harvey EL, Glenny AM, Kirk SF, Summerbell CD. Effective professional practice: protocol for a systematic review of health professional management of obesity. Journal of Human Nutrition and Dietetics 1998;11(3):243-7. Inappropriate Study Design

(113) Haynos AF, O'Donohue WT. Universal childhood and adolescent obesity prevention programs: review and critical analysis. Clin Psychol Rev 2012 July;32(5):383-99. Inappropriate Study Design

(114) Hebden L, Chey T, Allman-Farinelli M. Lifestyle intervention for preventing weight gain in young adults: a systematic review and meta-analysis of RCTs. Obesity Reviews 2012 August;13(8):692-710. Inappropriate Population

(115) Hendrie GA, Brindal E, Corsini N, Gardner C, Baird D, Golley RK. Combined home and school obesity prevention interventions for children: what behavior change strategies and intervention characteristics are associated with effectiveness? Health Education and Behavior 2012;39(2):159-71. Inappropriate Study Design

(116) Henness S, Perry CM. Orlistat: a review of its use in the management of obesity. Drugs 2006;66(12):1625-56. Inappropriate Study Design, Inappropriate Intervention

(117) Hesketh KD, Campbell KJ. Interventions to Prevent Obesity in 0-5 Year Olds: An Updated Systematic Review of the Literature. Obesity 2010;18:S27-S35. Inappropriate Study Design, Inappropriate Population

(118) Heymsfield SB, van Mierlo CA, van der Knaap HC, Heo M, Frier H, I. Weight management using a meal replacement strategy: meta and pooling analysis from six studies. International Journal of Obesity 2003;27(5):537-49. Inappropriate Intervention, Inappropriate Population

(119) Hillier F, Pedley C, Summerbell C. Evidence-base for primary prevention of obesity in children and adolescents. Bundesgesundheitsblatt-Gesundheitsforschung-Gesundheitsschutz 2011;54(3):259-64. Inappropriate Study Design

(120) Hingle MD, O'Connor TM, Dave JM, Baranowski T. Parental involvement in interventions to improve child dietary intake: A systematic review. Preventive Medicine 2010;51(2):103-11. Inappropriate Outcomes, Inappropriate Intervention

(121) Ho M, Garnett SP, Baur L, Burrows T, Stewart L, Neve M, Collins C. Effectiveness of lifestyle interventions in child obesity: systematic review with meta-analysis. Pediatrics 2012 December;130(6):e1647-e1671. Inappropriate Intervention

(122) Hoehner CM, Soares J, Perez DP, Ribeiro IC, Joshu CE, Pratt M, Legetic BD, Malta DC, Matsudo VR, Ramos LR, Simoes EJ, Brownson RC. Physical activity interventions in Latin America: a systematic review. American Journal of Preventive Medicine 2008;34(3):224-33. Inappropriate Study Design

(123) Hosking J, Macmillan A, Connor J, Bullen C, Ameratunga S. Organisational travel plans for improving health. Cochrane Database of Systematic Reviews 2010 January 1;2010:Art. Inappropriate Study Design, Inappropriate Intervention

(124) Hughes AR, Reilly JJ. Disease management programs targeting obesity in children - Setting the scene for wellness in the future. Disease Management & Health Outcomes 2008;16(4):255-66. Inappropriate Study Design

(125) Jacobson D, Gance-Cleveland B. A systematic review of primary healthcare provider education and training using the Chronic Care Model for Childhood Obesity. Obesity Reviews 2011;12(501):e244-e256. Inappropriate Study Design, Inappropriate Outcomes

(126) Jago R, Baranowski T. Non-curricular approaches for increasing physical activity in youth: a review. Preventive Medicine 2004;39(1):157-63. Inappropriate Study Design, Inappropriate Outcomes

(127) Jaime PC, Lock K. Do school based food and nutrition policies improve diet and reduce obesity? Preventive Medicine 2009;48(1):45-53. Inappropriate Study Design, Inappropriate Intervention

(128) Jakicic JM, Tate DF, Lang W, Davis KK, Polzien K, Rickman AD, Erickson K, Neiberg RH, Finkelstein EA. Effect of a stepped-care intervention approach on weight loss in adults: a randomized clinical trial. JAMA 2012;307(24):2617-26. Inappropriate Study Design, Inappropriate Population

(129) Jelalian E, Saelens BE. Empirically supported treatments in pediatric psychology: pediatric obesity. J Pediatr Psychol 1999 June;24(3):223-48. Inappropriate Study Design

(130) Jelalian E, Wember YM, Bungeroth H, Birmaher V. Practitioner review: bridging the gap between research and clinical practice in pediatric obesity. J Child Psychol Psychiatry 2007 February;48(2):115-27. Inappropriate Study Design

(131) Jerum A, Melnyk BM. Effectiveness of interventions to prevent obesity and obesity-related complications in children and adolescents. Pediatric Nursing 2001;27(6):606-10. Inappropriate Study Design

(132) Johnson ST, Newton AS, Chopra M, Buckingham J, Huang TTK, Franks PW, Jetha MM, Ball GDC. In search of quality evidence for lifestyle management and glycemic control in children and adolescents with type 2 diabetes: A systematic review. Bmc Pediatrics 2010;10. Inappropriate Population, Inappropriate Study Design

(133) Kamath CC, Vickers KS, Ehrlich A, McGovern L, Johnson J, Singhal V, Paulo R, Hettinger A, Erwin PJ, Montori VM. Behavioral Interventions to Prevent Childhood Obesity: A Systematic Review and Meta-analyses of Randomized Trials. Journal of Clinical Endocrinology & Metabolism 2008;93(12):4606-15. Inappropriate Population, Inappropriate Intervention

(134) Kanekar A, Sharma M. Meta-analysis of school-based childhood obesity interventions in the U.K. and U.S. Int Q Community Health Educ 2008;29(3):241-56. Inappropriate Population

(135) Katz DL, O'Connell M, Njike VY, Yeh MC, Nawaz H. Strategies for the prevention and control of obesity in the school setting: systematic review and meta-analysis. International Journal of Obesity 2008;32(12):1780-9. Inappropriate Intervention, Inappropriate Population

(136) Katz DL. School-Based Interventions for Health Promotion and Weight Control: Not Just Waiting on the World to Change. Annual Review of Public Health 2009;30:253-72. Inappropriate Study Design

(137) Kelley GA, Kelley KS. Aerobic exercise and lipids and lipoproteins in children and adolescents: A meta-analysis of randomized controlled trials. Atherosclerosis 2007;191(2):447-53. Inappropriate Outcomes

(138) Kelley GA, Kelley KS. Effects of aerobic exercise on non-high-density lipoprotein cholesterol in children and adolescents: a meta-analysis of randomized controlled trials. Progress in Cardiovascular Nursing 2008;23(3):128-32. Inappropriate Population, Inappropriate Outcomes

(139) Kelley GA, Kelley KS. Impact of progressive resistance training on lipids and lipoproteins in adults: a meta-analysis of randomized controlled trials. Preventive Medicine 2009;48 (1):9-19. Inappropriate Population, Inappropriate Outcomes

(140) Kelly KP, Kirschenbaum DS. Immersion treatment of childhood and adolescent obesity: the first review of a promising intervention. Obesity Reviews 2011;12(1):37-49. Inappropriate Intervention

(141) Kelly SA, Melnyk BM. Systematic review of multicomponent interventions with overweight middle adolescents: Implications for clinical practice and research. Worldviews on Evidence-Based Nursing 2008;5(3):113-35. Inappropriate Study Design, Inappropriate Comparison Group

(142) Kesten JM, Griffiths PL, Cameron N. A systematic review to determine the effectiveness of interventions designed to prevent overweight and obesity in pre-adolescent girls. Obesity Reviews 2011;12(12):997-1021. Inappropriate Population, Inappropriate Study Design

(143) Ketola E, Sipila R, Makela M. Effectiveness of individual lifestyle interventions in reducing cardiovascular disease and risk factors. Annals of Medicine 2000;32(4):239-51. Inappropriate Population

(144) Khambalia AZ, Dickinson S, Hardy LL, Gill T, Baur LA. A synthesis of existing systematic reviews and meta-analyses of school-based behavioural interventions for controlling and preventing obesity. Obesity Reviews 2012;13(3):214-33. Inappropriate Study Design

(145) Kiess W, Bottner A, Raile K, Kapellen T, Muller G, Galler A, Paschke R, Wabitsch M. Type 2 diabetes mellitus in children and adolescents: A review from a European perspective. Hormone Research 2003;59:77-84. Inappropriate Study Design, Inappropriate Outcomes

(146) Kilpelainen TO, Qi L, Brage S, Sharp SJ, Sonestedt E, Demerath E, Ahmad T, Mora S, Kaakinen M, Sandholt CH, Holzapfel C, Autenrieth CS, Hypponen E, Cauchi S, He MA, Kutalik Z, Kumari M, Stancakova A, Meidtner K, Balkau B, Tan JT, Mangino M, Timpson NJ, Song YQ, Zillikens MC et al. Physical Activity Attenuates the Influence of FTO Variants on Obesity Risk: A Meta-Analysis of 218,166 Adults and 19,268 Children. Plos Medicine 2011;8(11). Inappropriate Outcomes, Inappropriate Study Design

(147) Kim Y, Lee S. Physical activity and abdominal obesity in youth. Appl Physiol Nutr Metab 2009 August;34(4):571-81. Inappropriate Study Design

(148) Kirk S, Scott BJ, Daniels SR. Pediatric obesity epidemic: treatment options. Journal of the American Dietetic Association 2005 May;105(5 Suppl 1):S44-S51. Inappropriate Study Design

(149) Klesges LM, Williams NA, Davis KS, Buscemi J, Kitzmann KM. External Validity Reporting in Behavioral Treatment of Childhood Obesity A Systematic Review. American Journal of Preventive Medicine 2012;42(2):185-92. Inappropriate Study Design

(150) Knowlden AP, Sharma M. Systematic review of family and home-based interventions targeting paediatric overweight and obesity. Obesity Reviews 2012 June;13(6):499-508. Inappropriate Study Design

(151) Kriemler S, Meyer U, Martin E, van Sluijs EMF, Andersen LB, Martin BW. Effect of school-based interventions on physical activity and fitness in children and adolescents: a review of reviews and systematic update. British Journal of Sports Medicine 2011;45(11):923-30. Inappropriate Study Design, Inappropriate Outcomes

(152) Krishnaswami J, Martinson M, Wakimoto p, Anglemeyer A. Community-Engaged Interventions on Diet, Activity, and Weight Outcomes in U.S. Schools A Systematic Review. American Journal of Preventive Medicine 2012;43(1):81-91. Inappropriate Population

(153) Kropski JA, Keckley PH, Jensen GL. School-based obesity prevention programs: An evidence-based review. Obesity 2008;16(5):1009-18. Inappropriate Study Design

(154) Kuhl ES, Clifford LM, Stark LJ. Obesity in Preschoolers: Behavioral Correlates and Directions for Treatment. Obesity 2012;20(1):3-29. Inappropriate Study Design

(155) Laframboise MA, deGraauw C. The effects of aerobic physical activity on adiposity in school-aged children and youth: a systematic review of randomized controlled trials. Journal of the Canadian Chiropractic Association 2011 December;55(4):256-68. Inappropriate Population, Inappropriate Study Design

(156) Lambiase M. Treating pediatric overweight through reductions in sedentary behavior: a review of the literature. J Pediatr Health Care 2009 January;23(1):29-36. Inappropriate Study Design, Inappropriate Outcomes

(157) LaMonte MJ, Yanowitz FG. Aerobic exercise for lowering blood pressure: a metaanalysis. Clinical Journal of Sport Medicine 2002 November;12(6):407. Inappropriate Study Design

(158) Larson-Meyer DE. Effect of postpartum exercise on mothers and their offspring: a review of the literature. Obes Res 2002 August;10(8):841-53. Inappropriate Study Design, Inappropriate Population

(159) Laska MN, Pelletier JE, Larson NI, Story M. Interventions for Weight Gain Prevention During the Transition to Young Adulthood: A Review of the Literature. Journal of Adolescent Health 2012;50(4):324-33. Inappropriate Study Design

(160) Lavelle HV, Mackay DF, Pell JP. Systematic review and meta-analysis of school-based interventions to reduce body mass index. Journal of Public Health 2012;34(3):360-9. Inappropriate Population

(161) Lawlor DA, Smith GD. Early life determinants of adult blood pressure. Curr Opin Nephrol Hypertens 2005 May;14(3):259-64. Inappropriate Study Design, Inappropriate Population

(162) Leclerc KM. The role of exercise in reducing coronary heart disease and associated risk factors. Journal of the Oklahoma State Medical Association 1992 June;85(6):283-90. Inappropriate Study Design, Inappropriate Population

(163) LeMura LM, Maziekas MT. Factors that alter body fat, body mass, and fat-free mass in pediatric obesity. Medicine and Science in Sports and Exercise 2002;34(3):487-96. Inappropriate Study Design

(164) Lerro CC, McGlynn KA, Cook MB. A systematic review and meta-analysis of the relationship between body size and testicular cancer. British Journal of Cancer 2010;103(9):1467-74. Inappropriate Population, Inappropriate Study Design

(165) Leung MM, Agaronov A, Grytsenko K, Yeh MC. Intervening to Reduce Sedentary Behaviors and Childhood Obesity among School-Age Youth: A Systematic Review of Randomized Trials. J Obes 2012;2012:685430. doi: 10.1155/2012/685430. Epub;%2011 Oct 24.:685430. Inappropriate Study Design

(166) Li M, Li S, Baur LA, Huxley RR. A systematic review of school-based intervention studies for the prevention or reduction of excess weight among Chinese children and adolescents. Obesity Reviews 2008;9(6):548-59. Inappropriate Study Design

(167) Lissau I. Prevention of overweight in the school arena. Acta Paediatrica 2007;96(Supplement 454):12-8. Inappropriate Study Design

(168) Livesey G. Fructose, Obesity, and Related Epidemiology. Critical Reviews in Food Science and Nutrition 2010;50:26-8. Inappropriate Study Design

(169) Loomba R, Sirlin CB, Schwimmer JB, Lavine JE. Advances in pediatric nonalcoholic fatty liver disease. Hepatology 2009 October;50(4):1282-93. Inappropriate Study Design, Inappropriate Outcomes

(170) Lopez L, Audisio Y, Berra S. Effectiveness of population-based interventions on the prevention of overweight in children and adolescents. Medicina Clinica 2010;135(10):462-9. Inappropriate Study Design

(171) Loveman E, Frampton GK, Shepherd J, Picot J, Cooper K, Bryant J, Welch K, Clegg A. The clinical effectiveness and cost-effectiveness of long-term weight management schemes for adults: a systematic review. Health Technolology Assessment 2011 January;15(2):1-182. Inappropriate Study Design, Inappropriate Population

(172) Luckner H, Moss JR, Gericke CA. Effectiveness of interventions to promote healthy weight in general populations of children and adults: a meta-analysis. European Journal of Public Health 2012;22(4):491-7. Inappropriate Population

(173) Lyn R, McCarty F. Interpret results with caution. Canadian Medical Association Journal 2009 June 23;180(13):1330. Inappropriate Study Design

(174) Maniccia DM, Davison KK, Marshall SJ, Manganello JA, Dennison BA. A Meta-analysis of Interventions That Target Children's Screen Time for Reduction. Pediatrics 2011;128(1):E193-E210. Inappropriate Outcomes

(175) Martin A, Sanderson K, Cocker F. Meta-analysis of the effects of health promotion intervention in the workplace on depression and anxiety symptoms. Scandinavian Journal of Work, Environment and Health 2009;35(1):7-18. Inappropriate Population, Inappropriate Outcomes

(176) Martin S. [Nonpharmacological diabetes therapy]. Med Klin (Munich) 2006 December 15;101(12):973-89. Inappropriate Study Design, Inappropriate Population

(177) McCall A, Raj R. Exercise for prevention of obesity and diabetes in children and adolescents. Clinical Sports Medicine 2009 July;28(3):393-421. Inappropriate Study Design

(178) McCrory MA. Does dieting during lactation put infant growth at risk? Nutrition Reviews 2001 January;59(1 Pt 1):18-21. Inappropriate Study Design, Inappropriate Population

(179) Medina-Blanco RI, Jimenez-Cruz A, Perez-Morales ME, Armendariz-Anguiano AL, Bacardi-Gascon M. Intervention Programs to Promote Physical Activity in School Children: Systematic Review. Nutricion Hospitalaria 2011;26(2):265-70. Inappropriate Outcomes, Inappropriate Study Design

(180) Mekala KC, Tritos NA. Effects of recombinant human growth hormone therapy in obesity in adults: a metaanalysis. Journal of Clinical Endocrinology and Metabolism 2009;94(1):130-7. Inappropriate Population, Inappropriate Intervention

(181) Metcalf B, Henley W, Wilkin T. Effectiveness of intervention on physical activity of children: systematic review and meta-analysis of controlled trials with objectively measured outcomes (EarlyBird 54). British Medical Journal 2012;345. Inappropriate Outcomes, Inappropriate Study Design

(182) Meyers AW, Graves TJ, Whelan JP, Barclay DR. An evaluation of a television-delivered behavioural weight loss program: are the ratings acceptable? Journal of Consulting and Clinical Psychology 1996;64(1):172-8. Inappropriate Study Design

(183) Middleton p, Crowther CA, Simmonds L, Muller p. Different intensities of glycaemic control for pregnant women with pre-existing diabetes. Cochrane Database of Systematic Reviews: Reviews 2010;Issue 9. Inappropriate Population, Inappropriate Outcomes

(184) Mobley CC. Lifestyle interventions for "diabesity": the state of the science. Compend Contin Educ Dent 2004 March;25(3):207-2, 214. Inappropriate Study Design

(185) Monasta L, Batty GD, Cattaneo A, Lutje V, Ronfani L, van Lenthe FJ, Brug J. Early-life determinants of overweight and obesity: a review of systematic reviews. Obesity Reviews 2010;11(10):695-708. Inappropriate Study Design

(186) Monasta L, Batty GD, Macaluso A, Ronfani L, Lutje V, Bavcar A, van Lenthe FJ, Brug J, Cattaneo A. Interventions for the prevention of overweight and obesity in preschool children: a systematic review of randomized controlled trials. Obesity Reviews 2011;12(501):e107-e118. Inappropriate Study Design

(187) Moxley RT, Ashwal S, Pandya S, Connolly A, Florence J, Mathews K, Baumbach L, McDonald C, Sussman M, Wade C. Practice parameter: corticosteroid treatment of Duchenne dystrophy. Report of the Quality Standards Subcommittee of the American Academy of Neurology and the Practice Committee of the Child Neurology Society. Neurology 2005;64(1):13-20. Inappropriate Study Design, Inappropriate Intervention

(188) Mughal MZ, Khadilkar AV. The accrual of bone mass during childhood and puberty. Curr Opin Endocrinol Diabetes Obes 2011 February;18(1):28-32. Inappropriate Study Design, Inappropriate Outcomes

(189) Muktabhant B, Lumbiganon p, Ngamjarus C, Dowswell T. Interventions for preventing excessive weight gain during pregnancy. Cochrane Database of Systematic Reviews 2012;(4). Inappropriate Population

(190) Mulholland Y, Nicokavoura E, Broom J, Rolland C. Very-low-energy diets and morbidity: a systematic review of longer-term evidence. British Journal of Nutrition 2012;108 (5):832-51. Inappropriate Study Design, Inappropriate Intervention

(191) Nguyen B, Kornman KP, Baur LA. A review of electronic interventions for prevention and treatment of overweight and obesity in young people. Obesity Reviews 2011;12(501):e298-e314. Inappropriate Intervention

(192) NHS Centre for Reviews and Dissemination. Systematic review of interventions in the treatment and prevention of obesity. Report. 1997. Inappropriate Study Design

(193) Nichols MS, Swinburn BA. Selection of priority groups for obesity prevention: current approaches and development of an evidence-informed framework. Obesity Reviews 2010;11(10):731-9. Inappropriate Study Design, Inappropriate Outcomes

(194) Niemeier BS, Hektner JM, Enger KB. Parent participation in weight-related health interventions for children and adolescents: A systematic review and meta-analysis. Preventive Medicine 2012;55(1):3-13. Inappropriate Population

(195) Nikander R, nen H, Heinonen A, Daly RM, Uusi-Rasi K, Kannus p. Targeted exercise against osteoporosis: A systematic review and meta-analysis for optimising bone strength throughout life. BMC Medicine 2010 January 1;8:47. Inappropriate Outcomes

(196) Nixon CA, Moore HJ, Douthwaite W, Gibson EL, Vogele C, Kreichauf S, Wildgruber A, Manios Y, Summerbell CD, ToyBox-study Group. Identifying effective behavioural models and behaviour change strategies underpinning preschool- and school-based obesity prevention interventions aimed at 4-6-year-olds: a systematic review. Obesity Reviews 2012;13(Supplement 1):106-17. Inappropriate Study Design

(197) O'Connor TM, Jago R, Baranowski T. Engaging Parents to Increase Youth Physical Activity A Systematic Review. American Journal of Preventive Medicine 2009;37(2):141-9. Inappropriate Study Design, Inappropriate Population

(198) O'Meara S, Glenny AM, Sheldon T, Melville A, Wilson C. Systematic review of the effectiveness of interventions used in the management of obesity. Journal of Human Nutrition and Dietetics 1998;11(3):203-6. Inappropriate Study Design

(199) Ohkawara K, Tanaka S, Miyachi M, Ishikawa-Takata K, Tabata I. A dose-response relation between aerobic exercise and visceral fat reduction: systematic review of clinical trials. International Journal of Obesity (London) 2007 December;31(12):1786-97. Inappropriate Population

(200) Oostdam N, van Poppel MNM, Wouters MGAJ, van Mechelen W. Interventions for Preventing Gestational Diabetes Mellitus: A Systematic Review and Meta-Analysis. Journal of Womens Health 2011;20(10):1551-63. Inappropriate Outcomes, Inappropriate Population

(201) Oteng-Ntim E, Varma R, Croker H, Poston L, Doyle p. Lifestyle interventions for overweight and obese pregnant women to improve pregnancy outcome: Systematic review and meta-analysis. BMC Medicine 2012 January 1;10. Inappropriate Population

(202) Oude Luttikhuis H, Baur L, Jansen H, Shrewsbury VA, O'Malley C, Stolk RP, Summerbell CD. Interventions for treating obesity in children. Cochrane Database of Systematic Reviews 2009;(1). Inappropriate Study Design

(203) Palmer MA, Capra S, Baines SK. Association between eating frequency, weight, and health. Nutrition Reviews 2009 July;67(7):379-90. Inappropriate Study Design, Inappropriate Intervention

(204) Pate RR, O'Neill JR. After-school interventions to increase physical activity among youth. British Journal of Sports Medicine 2009 January;43(1):14-8. Inappropriate Study Design, Inappropriate Outcomes

(205) Peng LJ, Wang JY, Li F. Weight reduction for non-alcoholic fatty liver disease. Cochrane Database of Systematic Reviews 2011;(6). Inappropriate Population

(206) Perez-Morales ME, Bacardi-Gascon M, Jimenez-Cruz A, Armendariz-Anguiano A. Randomized controlled school based interventions to prevent childhood obesity: systematic review from 2006 to 2009. Archivos Latinoamericanos de Nutricion 2009;59(3):253-9. Inappropriate Study Design

(207) Perez-Rodrigo C, Aranceta BJ, Serra ML, Moreno B, Delgado RA. Epidemiology of obesity in Spain. Dietary guidelines and strategies for prevention. International Journal Vitamin Nutrition Reseach 2006 July;76(4):163-71. Inappropriate Study Design, Inappropriate Intervention

(208) Peterson KE, Fox MK. Addressing the epidemic of childhood obesity through school-based interventions: what has been done and where do we go from here? Journal of Law and Medical Ethics 2007;35(1):113-30. Inappropriate Study Design

(209) Phillips J, Phillips PJ. Children get type 2 diabetes too. Aust Fam Physician 2009 September;38(9):699-703. Inappropriate Study Design

(210) Pluim BM, Staal JB, Marks BL, Miller S, Miley D. Health benefits of tennis. British Journal of Sports Medicine 2007;41(11):760-8. Inappropriate Study Design, Inappropriate Population

(211) Pocock M, Trivedi D, Wills W, Bunn F, Magnusson J. Parental perceptions regarding healthy behaviours for preventing overweight and obesity in young children: a systematic review of qualitative studies. Obesity Reviews 2010;11(5):338-53. Inappropriate Outcomes, Inappropriate Study Design

(212) Poobalan AS, Aucott LS, Precious E, Crombie IK, Smith WC. Weight loss interventions in young people (18 to 25 year olds): a systematic review. Obesity Reviews 2010;11(8):580-92. Inappropriate Population

(213) Quinn SM, Baur LA, Garnett SP, Cowell CT. Treatment of clinical insulin resistance in children: a systematic review. Obesity Reviews 2010;11(10):722-30. Inappropriate Intervention

(214) Rafiq N, Younossi ZM. Effects of weight loss on nonalcoholic fatty liver disease. Semin Liver Dis 2008 November;28(4):427-33. Inappropriate Study Design

(215) Reilly JJ, McDowell ZC. Physical activity interventions in the prevention and treatment of paediatric obesity: systematic review and critical appraisal. Proceedings of the Nutrition Society 2003;62(3):611-9. Inappropriate Study Design

(216) Reilly JJ. Obesity in childhood and adolescence: evidence based clinical and public health perspectives. Postgraduate Medical Journal 2006;82(969):429-37. Inappropriate Study Design

(217) Reilly JJ. Evidence-Based Obesity Prevention in Childhood and Adolescence: Critique of Recent Etiological Studies, Preventive Interventions, and Policies. Advances in Nutrition 2012;3(4):636S-41S. Inappropriate Study Design

(218) Reinehr T. Effectiveness of lifestyle intervention in overweight children. Proceedings of the Nutrition Society 2011 November;70(4):494-505. Inappropriate Study Design

(219) Renders CM, Delemarre-van de Waal HA, Dekker JM, Hirasing RA. [Insulin resistance and diabetes type 2 in overweight children]. Ned Tijdschr Geneeskd 2003 October 18;147(42):2060-3. Inappropriate Study Design, Inappropriate Outcomes

(220) Ritchie LD, Crawford PB, Hoelscher DM, Sothern MS. Position of the American Dietetic Association: Individual-, family-, school-, and community-based interventions for pediatric overweight. Journal of the American Dietetic Association 2006;106(6):925-45. Inappropriate Study Design

(221) Robinson TN. Television viewing and childhood obesity. Pediatric Clinics of North America 2001 August;48(4):1017-25. Inappropriate Study Design

(222) Robinson WR, Poole C, Godley PA. Systematic review of prostate cancer's association with body size in childhood and young adulthood. Cancer Causes & Control 2008;19(8):793-803. Inappropriate Study Design, Inappropriate Population

(223) Ronnberg AK, Nilsson K. Interventions during pregnancy to reduce excessive gestational weight gain: a systematic review assessing current clinical evidence using the Grading of Recommendations, Assessment, Development and Evaluation (GRADE) system. BJOG 2010 October;117(11):1327-34. Inappropriate Study Design, Inappropriate Population

(224) Roux L, Kuntz KM, Donaldson C, Goldie SJ. Economic evaluation of weight loss interventions in overweight and obese women. Obesity 2006;14(6):1093-106. Inappropriate Study Design, Inappropriate Population

(225) Saavedra JM, Escalante Y, Garcia-Hermoso A. Improvement of aerobic fitness in obese children: a meta-analysis. International Journal of Pediatric Obesity 2011;6(3-4):169-77. Inappropriate Outcomes

(226) Saguil A, Stephens M. Interventions to Prevent Childhood Obesity. American Family Physician 2012;86(1):30-2. Inappropriate Study Design

(227) Sales S, Walker N. A systematic review of the effectiveness of weight management interventions in adults with learning disabilities. Journal of Human Nutrition & Dietetics 2011 June;24(3):303. Inappropriate Study Design, Inappropriate Population

(228) Salmon J, Booth ML, Phongsavan p, Murphy N, Timperio A. Promoting physical activity participation among children and adolescents. Epidemiologic Reviews 2007;29:144-59. Inappropriate Study Design, Inappropriate Outcomes

(229) Salmon J, Brown H, Hume C. Effects of strategies to promote children's physical activity on potential mediators. International Journal of Obesity 2009;33:S66-S73. Inappropriate Study Design, Inappropriate Outcomes

(230) Sargent GM, Pilotto LS, Baur LA. Components of primary care interventions to treat childhood overweight and obesity: a systematic review of effect. Obesity Reviews 2011;12(501):e219-e235. Inappropriate Study Design

(231) Sbruzzi G, Eibel B, Barbiero SM, Petkowicz RO, Ribeiro RA, Cesa CC, Martins CC, Marobin R, Schaan CW, Souza WB, Schaan BD, Pellanda LC. Educational interventions in childhood obesity: A systematic review with meta-analysis of randomized clinical trials. Preventive Medicine 2013 February 27;(13):10. Inappropriate Intervention

(232) Scheen AJ, Rorive M, Letiexhe M. [Physical exercise for preventing obesity, promoting weight loss and maintaining weight management]. Rev Med Liege 2001 April;56(4):244-7. Inappropriate Study Design

(233) Schmidt ME, Haines J, O'Brien A, McDonald J, Price S, Sherry B, Taveras EM. Systematic Review of Effective Strategies for Reducing Screen Time Among Young Children. Obesity 2012;20(7):1338-54. Inappropriate Intervention

(234) Schmitt NM, Nicholson WK, Schmitt J. The association of pregnancy and the development of obesity - results of a systematic review and meta-analysis on the natural history of postpartum weight retention. International Journal of Obesity 2007;31(11):1642-51. Inappropriate Population, Inappropriate Intervention

(235) Schwartz RS, Jaeger LF, Veith RC, Lakshminarayan S. The effect of diet or exercise on plasma norepinephrine kinetics in moderately obese young men. Int J Obes 1990 January;14(1):1-11. Inappropriate Study Design, Inappropriate Population

(236) Scrutinio D, Bellotto F, Lagioia R, Passantino A. Physical activity for coronary heart disease: cardioprotective mechanisms and effects on prognosis. Monaldi Arch Chest Dis 2005 June;64(2):77-87. Inappropriate Study Design

(237) Seo DC, Sa J. A Meta-Analysis of Obesity Interventions Among US Minority Children. Journal of Adolescent Health 2010;46(4):309-23. Inappropriate Comparison Group

(238) Sharma M. School-based interventions for childhood and adolescent obesity. Obesity Reviews 2006 August;7(3):261-9. Inappropriate Study Design

(239) Sharma M. Dietary education in school-based childhood obesity prevention programs. Advances in Nutrition 2011 March;2(2):207S-16S. Inappropriate Study Design, Inappropriate Intervention

(240) Shepherd J, Harden A, Rees R, Brunton G, Garcia J, Oliver S, Oakley A. Young people and healthy eating: a systematic review of research on barriers and facilitators. Report. 2002. Inappropriate Study Design, Inappropriate Intervention

(241) Sherry B. Food behaviors and other strategies to prevent and treat pediatric overweight. International Journal of Obesity (London) 2005 September;29 Suppl 2:S116-26.:S116-S126. Inappropriate Study Design

(242) Shin L, Bregman H, Frazier J, Noyes N. An overview of obesity in children with psychiatric disorders taking atypical antipsychotics. Harv Rev Psychiatry 2008;16(2):69-79. Inappropriate Study Design

(243) Silveira JAC, Taddei JAAC, Guerra PH, Nobre MRC. Effectiveness of school-based nutrition education interventions to prevent and reduce excessive weight gain in children and adolescents: a systematic review. Jornal de Pediatria 2011;87(5):382-92. Inappropriate Outcomes, Inappropriate Intervention

(244) Skouteris H, McCabe M, Swinburn B, Newgreen V, Sacher p, Chadwick p. Parental influence and obesity prevention in pre-schoolers: a systematic review of interventions. Obesity Reviews 2011;12(5):315-28. Inappropriate Study Design

(245) Small L, Anderson D, Melnyk BM. Prevention and early treatment of overweight and obesity in young children: a critical review and appraisal of the evidence. Pediatric Nursing 2007;33(2):149-52, 155. Inappropriate Study Design

(246) Smith AJ, Skow A, Bodurtha J, Kinra S. Health Information Technology in Screening and Treatment of Child Obesity: A Systematic Review. Pediatrics 2013;131(3):E894-E902. Inappropriate Study Design, Inappropriate Intervention

(247) Snethen JA, Broome ME, Cashin SE. Effective weight loss for overweight children: a meta-analysis of intervention studies. Journal of Pediatric Nursing 2006 February;21(1):45-56. Inappropriate Study Design, Inappropriate Intervention

(248) Sommer C, Hauser W, Burgmer M, Engelhardt R, Gerhold K, Petzke F, Schmidt-Wilcke T, Spath M, Tolle T, Uceyler N, Wang H, Winkelmann A, Thieme K. [Etiology and pathophysiology of fibromyalgia syndrome]. Schmerz 2012 June;26(3):259-67. Inappropriate Study Design, Inappropriate Population

(249) Spagnoli TD, Bioletti L, Bo C, Formigatti M. [TV, overweight and nutritional surveillance. Ads content, food intake and physical activity]. Ann Ig 2003 September;15(5):611-20. Inappropriate Study Design

(250) Staniford LJ, Breckon JD, Copeland RJ. Treatment of Childhood Obesity: A Systematic Review. Journal of Child and Family Studies 2012;21(4):545-64. Inappropriate Population

(251) Steyn NP, Lambert E, V, Tabana H. Nutrition interventions for the prevention of type 2 diabetes. Proceedings of the Nutrition Society 2009;68(1):55-70. Inappropriate Study Design, Inappropriate Intervention

(252) Stice E, Shaw H, Marti CN. A meta-analytic review of obesity prevention programs for children and adolescents: The skinny on interventions that work. Psychological Bulletin 2006;132(5):667-91. Inappropriate Population, Inappropriate Intervention

(253) Stone EJ, McKenzie TL, Welk GJ, Booth ML. Effects of physical activity interventions in youth. Review and synthesis. American Journal of Preventive Medicine 1998 November 1;15(4):298-315. Inappropriate Study Design, Inappropriate Outcomes

(254) Straker LM, Smith KL, Fenner AA, Kerr DA, McManus A, Davis MC, Fielding AM, Olds TS, Hagger MS, Smith AJ, Abbott RA. Rationale, design and methods for a staggered-entry, waitlist controlled clinical trial of the impact of a community-based, family-centred, multidisciplinary program focussed on activity, food and attitude habits (Curtin University's Activity, Food and Attitudes Program-CAFAP) among overweight adolescents. Bmc Public Health 2012;12. Inappropriate Study Design

(255) Streuling I, Beyerlein A, von Kries R. Can gestational weight gain be modified by increasing physical activity and diet counseling? A meta-analysis of interventional trials. American Journal of Clinical Nutrition 2010;92(4):678-87. Inappropriate Population, Inappropriate Intervention

(256) Stuart WP, Broome ME, Smith BA, Weaver M. An integrative review of interventions for adolescent weight loss. Journal of School Nursing 2005;21(2):77-85. Inappropriate Study Design

(257) Sui Z, Grivell RM, Dodd JM. Antenatal exercise to improve outcomes in overweight or obese women: a systematic review. Acta Obstetricia et Gynecologica Scandinavica 2012;91(5):538-45. Inappropriate Population

(258) Sung-Chan p, Sung YW, Zhao X, Brownson RC. Family-based models for childhood-obesity intervention: a systematic review of randomized controlled trials. Obesity Reviews 2013;14(4):265-78. Inappropriate Study Design, Inappropriate Outcomes

(259) Swanson M, Studts CR, Bardach SH, Bersamin A, Schoenberg NE. Intergenerational Energy Balance Interventions: A Systematic Literature Review. Health Education & Behavior 2011;38(2):171-97. Inappropriate Study Design

(260) Szczepaniak-Chichel L, Tykarski A. [Treatment of arterial hypertension in pregnancy in relation to current guidelines of the Polish Society of Arterial Hypertension from 2011]. Ginekol Pol 2012 October;83(10):778-83. Inappropriate Study Design, Inappropriate Population

(261) Tambalis K, Panagiotakos DB, Kavouras SA, Sidossis LS. Responses of blood lipids to aerobic, resistance, and combined aerobic with resistance exercise training: a systematic review of current evidence. Angiology 2009;60(5):614-32. Inappropriate Outcomes, Inappropriate Population

(262) Tanentsapf I, Heitmann BL, Adegboye ARA. Systematic review of clinical trials on dietary interventions to prevent excessive weight gain during pregnancy among normal weight, overweight and obese women. Bmc Pregnancy and Childbirth 2011;11. Inappropriate Population

(263) Taylor BJ, Heath ALM, Galland BC, Gray AR, Lawrence JA, Sayers RM, Dale K, Coppell KJ, Taylor RW. Prevention of Overweight in Infancy (POI.nz) study: a randomised controlled trial of sleep, food and activity interventions for preventing overweight from birth. Bmc Public Health 2011;11. Inappropriate Study Design

(264) Thangaratinam S, Rogozinska E, Jolly K, Glinkowski S, Duda W, Borowiack E, Roseboom T, Tomlinson J, Walczak J, Kunz R, Mol BW, Coomarasamy A, Khan KS. Interventions to reduce or prevent obesity in pregnant women: a systematic review. Health Technology Assessment 2012;16(31):1-+. Inappropriate Population

(265) Thomas H, Ciliska D, Micucci S, Wilson-Abra J, Dobbins M. Effectiveness of physical activity enhancement and obesity prevention programs in children and youth (Report). Hamilton, ON: Effective Public Health Practice Project (EPHPP); 2004 Jan 1. Inappropriate Study Design

(266) Thomas H. Obesity prevention programs for children and youth: why are their results so modest? Health Education Research 2006 December;21(6):783-95. Inappropriate Study Design

(267) Thomas J, Sutcliffe K, Harden A, Oakley A, Oliver S, Rees R, Brunton G, Kavanagh J. Children and healthy eating: a systematic review of barriers and facilitators. Report. 2003. Inappropriate Intervention

(268) Thompson S, Ekelund U, Jebb S, Lindroos AK, Mander A, Sharp S, Turner R, Wilks D. A proposed method of bias adjustment for meta-analyses of published observational studies. International Journal of Epidemiology 2011;40(3):765-77. Inappropriate Study Design

(269) Thomson CA, Ravia J. A Systematic Review of Behavioral Interventions to Promote Intake of Fruit and Vegetables. Journal of the American Dietetic Association 2011;111(10):1523-35. Inappropriate Outcomes, Inappropriate Population

(270) Tremblay MS, LeBlanc AG, Kho ME, Saunders TJ, Larouche R, Colley RC, Goldfield G, Gorber SC. Systematic review of sedentary behaviour and health indicators in school-aged children and youth. International Journal of Behavioral Nutrition and Physical Activity 2011;8. Inappropriate Intervention, Inappropriate Population

(271) Tsiros MD, Sinn N, Coates AM, Howe PRC, Buckley JD. Treatment of adolescent overweight and obesity. European Journal of Pediatrics 2008;167(1):9-16. Inappropriate Study Design

(272) Tsiros MD, Olds T, Buckley JD, Grimshaw p, Brennan L, Walkley J, Hills AP, Howe PR, Coates AM. Health-related quality of life in obese children and adolescents. International Journal of Obesity (London) 2009 April;33(4):387-400. Inappropriate Outcomes, Inappropriate Intervention

(273) Tur J, Alos M, Iglesias L, Luque L, Colom A, Escudero A, Martinez D, Pagan A, Ugarriza E, Frontera M, Nicola G, Palomero A, Tofe S, Urgeles JR, Barcelo MA, Couce M, De La Pena M, Fiol M, Cortes B, Teres E, Tumbarello A, Alvarez C, Salinas R, Pereg V, Gonzalez X et al. [TRAMOMTANA (Multidisciplinary treatment of morbid obesity: medication, behavioral therapy, nutritional support, and physical activity). From question to reality in an investigator-initiated clinical trial (II)]. Endocrinol Nutr 2011 June;58(6):299-307. Inappropriate Study Design

(274) Van Cauwenberghe E, Maes L, Spittaels H, van Lenthe FJ, Brug J, Oppert JM, De Bourdeaudhuij I. Effectiveness of school-based interventions in Europe to promote healthy nutrition in children and adolescents: systematic review of published and 'grey' literature. British Journal of Nutrition 2010;103(6):781-97. Inappropriate Intervention, Inappropriate Outcomes

(275) van Grieken A, Ezendam NPM, Paulis WD, van der Wouden JC, Raat H. Primary prevention of overweight in children and adolescents: a meta-analysis of the effectiveness of interventions aiming to decrease sedentary behaviour. International Journal of Behavioral Nutrition and Physical Activity 2012;9. Inappropriate Population

(276) Van Lippevelde W, Verloigne M, De Bourdeaudhuij I, Brug J, Bjelland M, Lien N, Maes L. Does parental involvement make a difference in school-based nutrition and physical activity interventions? A systematic review of randomized controlled trials. International Journal of Public Health 2012;57(4):673-8. Inappropriate Study Design

(277) van Sluijs EMF, Mcminn AM, Griffin S. Effectiveness of interventions to promote physical activity in children and adolescents: systematic review of controlled trials. British Medical Journal 2007;335(7622):703-7. Inappropriate Study Design, Inappropriate Outcomes

(278) van Sluijs EMF, Kriemler S, Mcminn AM. The effect of community and family interventions on young people's physical activity levels: a review of reviews and updated systematic review. British Journal of Sports Medicine 2011;45(11):914-22. Inappropriate Study Design

(279) van Stralen MM, Yildirim M, Velde SJT, Brug J, van Mechelen W, Chinapaw MJM. What works in school-based energy balance behaviour interventions and what does not? A systematic review of mediating mechanisms. International Journal of Obesity 2011;35(10):1251-65. Inappropriate Outcomes, Inappropriate Population

(280) Van Wijnen LG, Wendel-Vos GC, Wammes BM, Bemelmans WJ. The impact of school-based prevention of overweight on psychosocial well-being of children. Obesity Reviews 2009;10(3):298-312. Inappropriate Study Design, Inappropriate Outcomes

(281) van TE, Wilson B, Barry N, Ralph A, McNeill G, Graham W, Campbell D. Effectiveness of interventions to promote healthy eating in pregnant women and women of childbearing age: a review. Report. 1998. Inappropriate Intervention, Inappropriate Population

(282) Verstraeten R, Roberfroid D, Lachat C, Leroy JL, Holdsworth M, Maes L, Kolsteren PW. Effectiveness of preventive school-based obesity interventions in low- and middle-income countries: a systematic review. American Journal of Clinical Nutrition 2012;96(2):415-38. Inappropriate Population

(283) Viner RM, Hsia Y, Tomsic T, Wong IC. Efficacy and safety of anti-obesity drugs in children and adolescents: systematic review and meta-analysis. Obesity Reviews 2010;11(8):593-602. Inappropriate Intervention

(284) Wahi G, Parkin PC, Beyene J, Uleryk EM, Birken CS. Effectiveness of Interventions Aimed at Reducing Screen Time in Children A Systematic Review and Meta-analysis of Randomized Controlled Trials. Archives of Pediatrics & Adolescent Medicine 2011;165(11):979-86. Inappropriate Intervention, Inappropriate Population

(285) Wake M, Gold L, McCallum Z, Gerner B, Waters E. Economic evaluation of a primary care trial to reduce weight gain in overweight/obese children: the LEAP trial. Ambulatory Pediatrics 2008;8(5):336-41. Inappropriate Study Design

(286) Wake M, Baur LA, Gerner B, Gibbons K, Gold L, Gunn J, Levickis p, McCallum Z, Naughton G, Sanci L, Ukoumunne OC. Outcomes and costs of primary care surveillance and intervention for overweight or obese children: the LEAP 2 randomised controlled trial. British Medical Journal 2009 September 3;339:b3308. doi: 10.1136/bmj.b3308.:b3308. Inappropriate Study Design

(287) Wang LY, Yang Q, Lowry R, Wechsler H. Economic analysis of a school-based obesity prevention program. Obesity Research 2003;11(11):1313-24. Inappropriate Study Design, Inappropriate Outcomes

(288) Wang LY, Gutin B, Barbeau p, Moore JB, Hanes J, Johnson MH, Cavnar M, Thornburg J, Yin Z. Cost-effectiveness of a school-based obesity prevention program. Journal of School Health 2008;78(12):619-24. Inappropriate Study Design, Inappropriate Outcomes

(289) Wareham NJ, van Sluijs EM, Ekelund U. Physical activity and obesity prevention: a review of the current evidence. Proceedings of the Nutrition Society 2005 May;64(2):229-47. Inappropriate Study Design

(290) Watkins D, McCarron p, Murray L, Cran G, Boreham C, Robson p, McGartland C, Smith GD, Savage M. Trends in blood pressure over 10 years in adolescents: Analyses of cross sectional surveys in the Northern Ireland Young Hearts project. British Medical Journal 2004;329(7458):139-41. Inappropriate Study Design, Inappropriate Outcomes

(291) Watts K, Jones TW, Davis EA, Green D. Exercise training in obese children and adolescents: current concepts. Sports Medicine 2005;35(5):375-92. Inappropriate Study Design

(292) Weaver RG, Beets MW, Webster C, Beighle A, Huberty J. A Conceptual Model for Training After-School Program Staffers to Promote Physical Activity and Nutrition. Journal of School Health 2012;82(4):186-95. Inappropriate Study Design

(293) Weickert MO, Pfeiffer AF. [Preventing type 2 diabetes: what does dietary fiber achieve?]. MMW Fortschr Med 2005 April 28;147(17):28-30. Inappropriate Study Design, Inappropriate Intervention

(294) Whitlock EP, O'Connor EA, Williams SB, Beil TL, Lutz KW. Effectiveness of weight management programs in children and adolescents. Report. 2008. Inappropriate

Population

(295) Whitlock EP, O'Connor EA, Williams SB, Beil TL, Lutz KW. Effectiveness of primary care interventions for weight management in children and adolescents: an updated, targeted systematic review for the USPSTF. Report. 2010. Inappropriate Population

(296) Wilfley DE, Haymond M, Anderson B, Gunn S, Holden H, Jones M, Hwu K, McGirk S, Mckay S, Schreiner B, Cuttler L, Abrams E, Casey T, Dahms W, Drotar D, Huestis S, Levers-Landis C, McGuigan p, Sundararajan S, Geffner M, Chang N, Dreimane D, Halvorson M, Hernandez S, Kaufman F et al. Design of a family-based lifestyle intervention for youth with type 2 diabetes: the TODAY study The TODAY Study Group. International Journal of Obesity 2010;34(2):217-26. Inappropriate Study Design, Inappropriate Population

(297) Wilkin TJ. Can we modulate physical activity in children? No. International Journal of Obesity 2011;35(10):1270-6. Inappropriate Study Design

(298) Wilks DC, Sharp SJ, Ekelund U, Thompson SG, Mander AP, Turner RM, Jebb SA, Lindroos AK. Objectively Measured Physical Activity and Fat Mass in Children: A Bias-Adjusted Meta-Analysis of Prospective Studies. Plos One 2011;6(2). Inappropriate Study Design

(299) Williams DM, Matthews CE, Rutt C, Napolitano MA, Marcus BH. Interventions to increase walking behavior. Medicine and Science in Sports and Exercise 2008;40(7 Supplement):S567-S573. Inappropriate Population

(300) Wolfenden L, Wiggers J, d'Espaignet ET, Bell AC. How useful are systematic reviews of child obesity interventions? Obesity Reviews 2010;11(2):159-66. Inappropriate Study Design

(301) Wood PD. Impact of experimental manipulation of energy intake and expenditure on body composition. Critical Reviews in Food Science and Nutrition 1993;33(4-5):369-73. Inappropriate Study Design, Inappropriate Population

(302) Woodward-Lopez G, Kao J, Ritchie L.
To what extent have sweetened beverages contributed to the obesity epidemic? Public Health Nutrition 2011;14(3):499-509. Inappropriate Outcomes, Inappropriate Study Design

(303) Wortman J. Health promotion when the 'vaccine' does not work. Health Promotion Journal of Australia 2006 August;17(2):91-6. Inappropriate Study Design, Inappropriate Intervention

(304) Wuelling SM. Systematic review of interventions designed to reduce screen time in children and adolescents. United States -- Texas: The University of Texas School of Public Health; 2007. Inappropriate Study Design

(305) Yang L, Sahlqvist S, McMinn A, Griffin SJ, Ogilvie D. Interventions to promote cycling: systematic review. British Medical Journal 2010;341. Inappropriate Population, Inappropriate Outcomes

(306) Yildirim M, van Stralen MM, Chinapaw MJM, Brug J, van Mechelen W, Twisk JWR, Velde SJT. For whom and under what circumstances do school-based energy balance behavior interventions work? Systematic review on moderators. International Journal of Pediatric Obesity 2011;6(2-2):E46-E57. Inappropriate Study Design, Inappropriate Outcomes
